# An R Package for Computing Canadian Assessment of Physical Literacy (CAPL) scores and interpretations from raw data

**DOI:** 10.1371/journal.pone.0243841

**Published:** 2021-02-22

**Authors:** Joel D. Barnes, Michelle D. Guerrero

**Affiliations:** 1 Independent Researcher, Canada; 2 Children’s Hospital of Eastern Ontario Research Institute, Ottawa, Ontario, Canada; Southern Illinois University, UNITED STATES

## Abstract

The Canadian Assessment of Physical Literacy (CAPL) is the first comprehensive protocol designed to assess a child’s level of physical literacy. Current approaches to analyzing CAPL-2 raw data are tedious, inefficient, and/or can lead to computation errors. In this paper we introduce the capl R package (open source), designed to compute and visualize CAPL-2 scores and interpretations from raw data. The capl package takes advantage of the R environment to provide users with a fast, efficient, and reliable approach to analyzing their CAPL-2 raw data and a “quiet” user experience, whereby “noisy” error messages are suppressed via validation. We begin by discussing several preparatory steps that are required prior to using the capl package. These steps include preparing, formatting, and importing CAPL-2 raw data. We then use demo data to show that computing the CAPL-2 scores and interpretations is as simple as executing one line of code. This one line of code uses the main function in the capl package (get_capl()) to compute 40 variables within a matter of seconds. Next, we showcase the helper functions that are called within the main function to compute individual variables and scores for each test element within the four domains as well as an overall physical literacy score. Finally, we show how to visualize CAPL-2 results using the ggplot2 R package.

## 1. Introduction

Physical literacy is defined as “the motivation, confidence, physical competence, knowledge, and understanding to value and take responsibility for engagement in physical activities for life” [1]. It has been recognized as the foundation for lifelong healthy active living [2], and subsequently impacted the work of numerous sectors, including physical activity, sport, recreation, education, and public health [1]. Though the construct of physical literacy has gained significant attention in recent years [3–5], early advocates emphasized its importance and highlighted the need for a comprehensive and objective measurement of physical literacy as a means to understand the state of physical literacy in children, evaluate the effectiveness of physical activity programming initiatives, and increase the robustness of physical education assessment [6].

The Canadian Assessment of Physical Literacy (CAPL) was the first comprehensive protocol designed to assess a broad spectrum of skills and abilities that contribute to and characterize the physical literacy level of a participating child [2]. The CAPL was developed on the premise that a physically active child is more likely to possess adequate knowledge and understanding of physical activity, motivation and confidence, and physical competence than a physically inactive child. The first version of CAPL was developed and refined between 2009 and 2013 [7] and later revised (CAPL-2) in 2017. The CAPL-2 reflects revisions based on assessments of over 10,000 Canadian children and is the culmination of test development efforts, with input from well over 100 researchers and practitioners within related fields of study [8]. The CAPL-2 comprises four domains: physical competence, daily behaviour, motivation and confidence, and knowledge and understanding. Each domain consists of different test elements. These test elements can be scored and interpreted independently to provide an assessment of each attribute of physical literacy or can be combined to provide comprehensive scores for each domain. An overall physical literacy score can also be calculated using each of the four domain scores, with suggested interpretations – based on normative data from over 10,000 Canadian children [7] – by age and gender. Numerical CAPL-2 scores are assigned to one of four categories: beginning, progressing, achieving, and excelling. The beginning and progressing categories include children who have not yet achieved the optimal level of physical literacy, the achieving category identifies children who have achieved a score associated with sufficient physical literacy, and the excelling category reflects children with a high level of physical literacy.

The number of published research studies using the CAPL/CAPL-2 continues to grow. In Canada alone, 14 papers from the Royal Bank of Canada Learn to Play – Canadian Assessment of Physical Literacy study (RBC – Learn to Play CAPL) were published in a supplemental issue of BMC Public Health (bmcpublichealth.biomedcentral.com/articles/supplements/volume-18-supplement-2). Data in each paper included approximately 10,000 children aged 8 to 12 years, recruited from several provinces across Canada. The CAPL-2 manual and materials have been translated in five languages and have been used internationally [9].

In this paper we introduce the capl R package, designed to compute and visualize CAPL-2 scores and interpretations from raw data. R is a programming language and free software environment for statistical computing and graphics (https://www.r-project.org/about.html). R is widely used among statisticians and data analysts, and is among the top 10 most popular programming languages according to the TIOBE Programming Community index (www.tiobe.com/tiobe-index). The capl package is open source and was built to provide users with a fast, efficient, and reliable approach to analyzing CAPL-2 raw data. Currently, users can analyze CAPL-2 raw data either manually or through the CAPL-2 website (www.capl-eclp.ca). Manually analyzing CAPL-2 raw data requires users to navigate through approximately 60 variables and perform dozens of tedious calculations which are derived from different cut-off criteria, existing variables, and newly created variables. Hence, this method is often time-intensive and can lead to errors in scores and interpretations due to incorrect calculations or data entry errors. The data entry feature on the CAPL-2 website reduces user burden associated with data manipulation and analysis by computing scores and interpretations for the user. The primary disadvantage of this feature, however, is that only one participant’s data can be analyzed at one time, making this approach monotonous and time-intensive. As shown in Supplementary File A, users using the data entry feature on the CAPL website are required to enter the raw data of every test element for each participant. Another disadvantage associated with the website is that some users from academic institutions seeking to analyze their raw data via the website are often prohibited because of privacy and ethical concerns raised by institutional research ethics boards. The capl package was specifically designed to address these issues. As shown below, the capl package was developed to analyze raw data from hundreds and thousands of observations (i.e., participants) all at once, in one simple line of code. A number of helper functions in the package serve to validate the raw data in order to minimize errors and non-sensical scores and interpretations (see section 2.7.1).

## 2. Getting started

### 2.1 Installation

Users can download and install the most recent version of the capl package directly from GitHub (www.github.com/barnzilla/capl) using the devtools R package.

devtools::**install_github**(

    repo = "barnzilla/capl",

    upgrade = "never",

    build_vignettes = TRUE,

    force = TRUE

)

**library**(capl)

Once the capl package is loaded, any available tutorials for the package can be accessed by calling the browseVignettes() function.

**browseVignettes**("capl")

The name and description of each function included in the capl package is outlined in Table 1.

**Table 1:** Name and description of each function included in the capl package.

****Name****	****Description****
capitalize_character()	This function capitalizes a character vector.
capl_demo_data()	A dataset containing CAPL-2 demo raw data.
export_capl_data()	This function exports CAPL-2 data to an Excel workbook on a local computer.
get_24_hour_clock()	This function converts 12-hour clock values to 24-hour clock values.
get_adequacy_score()	This function computes an adequacy score (adequacy_score) for responses to items 2, 4 and 6 of the CSAPPA (Children’s Self-Perceptions of Adequacy in and Predilection for Physical Activity; Hay, 1992) Questionnaire as they appear in the CAPL-2 Questionnaire. This score is used to compute the motivation and confidence domain score (mc_score).
get_binary_score()	This function computes a binary score (0 = incorrect answer, 1 = correct answer) for a response to a questionnaire item based on the value(s) set as answer(s) to the item.
get_camsa_score()	This function selects the maximum CAMSA (Canadian Agility and Movement Skill Assessment) skill + time score for two trials (camsa_score) and then divides by 2.8 so that the score is out of 10. This score is used to compute the physical literacy score (pc_score).
get_camsa_skill_time_score()	This function computes the CAMSA (Canadian Agility and Movement Skill Assessment) skill + time score (e.g., camsa_skill_time_score1) for a given trial. This score is used to compute the CAMSA score (camsa_score).
get_camsa_time_score()	This function computes the CAMSA (Canadian Agility and Movement Skill Assessment) time score based on the time taken (in seconds) to complete a trial.
get_capl()	This function is the main function in the capl package. It is a wrapper function that calls all other capl functions to compute all CAPL-2 scores and interpretations from raw data at once. If required CAPL-2 variables are missing, the function will create the variables and set values for these variables to NA so the function can proceed.
get_capl_bar_plot()	This function renders a bar plot for a given CAPL-2 domain score, grouped by CAPL-2 interpretative categories.
get_capl_demo_data()	This function generates a data frame of CAPL-2 demo (fake) raw data containing the 60 required variables that the capl package needs to compute scores and interpretations.
get_capl_domain_status()	This function computes the status ("complete", "missing interpretation", "missing protocol" or "incomplete") of a CAPL domain (e.g., pc_status, db_status, mc_status, ku_status, capl_status).
get_capl_interpretation()	This function computes an age- and gender-specific CAPL-2 interpretation for a given CAPL-2 protocol or domain score (e.g., pc_interpretation).
get_capl_score()	This function computes an overall physical literacy score (capl_score) based on the physical competence (pc_score), daily behaviour (db_score), motivation and confidence (mc_score), and knowledge and understanding (ku_score) domain scores. If one of the scores is missing or invalid, a weighted score will be computed from the other three scores.
get_db_score()	This function computes a daily behaviour domain score (db_score) based on the step and self-reported physical activity scores. This score is used to compute the overall physical literacy score (capl_score).
get_fill_in_the_blanks_score()	This function computes a score (fill_in_the_blanks_score) for responses to the fill in the blanks items (story about Sally) in the CAPL-2 Questionnaire. This score is used to compute the knowledge and understanding domain score (ku_score).
get_intrinsic_motivation_score()	This function computes an intrinsic motivation score (intrinsic_motivation_score) for responses to items 1-3 of the the Behavioral Regulation in Exercise Questionnaire (BREQ) as they appear in the CAPL-2 Questionnaire. This score is used to compute the motivation and confidence domain score (mc_score).
get_ku_score()	This function computes a knowledge and understanding domain score (ku_score) based on the physical activity guideline (pa_guideline_score), cardiorespiratory fitness means (crf_means_score), muscular strength and endurance means (ms_score), sports skill (sports_skill_score) and fill in the blanks (fill_in_the_blanks_score) scores. If one of the scores is missing or invalid, a weighted domain score will be computed from the other four scores. This score is used to compute the overall physical literacy score (capl_score).
get_mc_score()	This function computes a motivation and confidence domain score (mc_score) based on the predilection (predilection_score), adequacy (adequacy_score), intrinsic motivation (intrinsic_motivation_score) and physical activity competence (pa_competence_score) scores. If one of the scores is missing or invalid, a weighted domain score will be computed from the other three scores. This score is used to compute the overall physical literacy score (capl_score).
get_missing_capl_variables()	This function adds required CAPL-2 variables (see Details for a full list) to a data frame of raw data if they are missing. When missing variables are added, the values for a given missing variable are set to NA. This function is called within get_capl() so that CAPL-2 score and interpretation computations will run without errors in the presence of missing variables.
get_pa_competence_score()	This function computes a physical activity competence score (pa_competence_score) for responses to items 4-6 of the the Behavioral Regulation in Exercise Questionnaire (BREQ) as they appear in the CAPL-2 Questionnaire. This score is used to compute the motivation and confidence domain score (mc_score).
get_pacer_20m_laps()	This function converts PACER (Progressive Aerobic Cardiovascular Endurance Run) shuttle run laps to their equivalent in 20-metre laps (pacer_laps_20m). If laps are already 20-metre laps, they are returned unless outside the valid range (1-229). This variable is used to compute the PACER score (pacer_score).
get_pacer_score()	This function computes a PACER (Progressive Aerobic Cardiovascular Endurance Run) score (pacer_score) based on the number of PACER laps run at a 20-metre distance. This score is used to compute the physical competence domain score variable (pc_score).
get_pc_score()	This function computes a physical competence domain score (pc_score) based on the PACER (Progressive Aerobic Cardiovascular Endurance Run), plank and CAMSA (Canadian Agility and Movement Skill Assessment) scores. If one protocol score is missing or invalid, a weighted domain score will be computed from the other two protocol scores. This score is used to compute the physical competence domain score (pc_score).
get_pedometer_wear_time()	This function computes pedometer wear time in decimal hours for a given day (e.g., wear_time1). This variable is used to compute the step_average variable and the step score (step_score).
get_plank_score()	This function computes a plank score (plank_score) based on the duration of time (in seconds) for which a plank is held. This score is used to compute the physical competence domain score (pc_score).
get_predilection_score()	This function computes a predilection score (predilection_score) for responses to items 1, 3 and 5 of the CSAPPA (Children’s Self-Perceptions of Adequacy in and Predilection for Physical Activity; Hay, 1992) Questionnaire as they appear in the CAPL-2 Questionnaire. This score is used to compute the motivation and confidence domain score (mc_score).
get_self_report_pa_score()	This function computes a score (self_report_pa_score) for a response to "During the past week (7 days), on how many days were you physically active for a total of at least 60 minutes per day? (all the time you spent in activities that increased your heart rate and made you breathe hard)?" in the CAPL-2 Questionnaire. This score is used to compute the daily behaviour domain score (db_score).
get_step_average()	This function computes the daily arithmetic mean of a week of steps taken as measured by a pedometer (step_average). This variable is used to compute the step score (step_score).
get_step_score()	This function computes a step score (step_score) based on the average daily steps taken as measured by a pedometer. This score is used to compute the daily behaviour domain score (db_score).
import_capl_data()	This function imports CAPL-2 data from an Excel workbook on a local computer.
rename_variable()	This function renames variables in a data frame.
validate_age()	This function checks whether an age is valid (numeric and between 8 and 12). CAPL-2 scores and interpretations are valid for children between the ages of 8 and 12 years.
validate_character()	This function checks whether a vector is a character and not of length zero or "".
validate_domain_score()	This function checks whether a CAPL-2 domain score is numeric and within a valid range.
validate_gender()	This function checks whether a vector can be classified as "girl" or "boy".
validate_integer()	This function checks whether a vector is an integer.
validate_number()	This function checks whether a vector is numeric.
validate_scale()	This function checks whether a vector for a given questionnaire item or scale is valid.
validate_steps()	This function checks whether daily steps as measured by a pedometer are valid. The variables from this function are used to compute step_average and the step score (step_score).

### 2.2 Importing raw data

Users must first import their raw data before using the capl package to compute CAPL-2 scores and interpretations. The import_capl_data() function enables users to import data from an Excel workbook into the R global environment.

data <- **import_capl_data**(

    file_path = "c:/users/joel/desktop/data.xlsx",

    sheet_name = "Sheet1"

)

### 2.3 Required variables

The capl package requires 60 variables in order to compute CAPL-2 scores and interpretations. Users can use the get_missing_capl_variables() function to retrieve a list of the required variables. The required variables are outlined in the Details section of the documentation.

?get_missing_capl_variables

○ age

○ gender

○ pacer_lap_distance

○ pacer_laps

○ plank_time

○ camsa_skill_score1

○ camsa_time1

○ camsa_skill_score2

○ camsa_time2

○ steps1

○ time_on1

○ time_off1

○ non_wear_time1

○ steps2

○ time_on2

○ time_off2

○ non_wear_time2

○ steps3

○ time_on3

○ time_off3

○ non_wear_time3

○ steps4

○ time_on4

○ time_off4

○ non_wear_time4

○ steps5

○ time_on5

○ time_off5

○ non_wear_time5

○ steps6

○ time_on6

○ time_off6

○ non_wear_time6

○ steps7

○ time_on7

○ time_off7

○ non_wear_time7

○ self_report_pa

○ csappa1

○ csappa2

○ csappa3

○ csappa4

○ csappa5

○ csappa6

○ why_active1

○ why_active2

○ why_active3

○ feelings_about_pa1

○ feelings_about_pa2

○ feelings_about_pa3

○ pa_guideline

○ crf_means

○ ms_means

○ sports_skill

○ pa_is

○ pa_is_also

○ improve

○ increase

○ when_cooling_down

○ heart_rate

### 2.4 Loading the pre-installed dataset

The capl package comes with a demo (fake) dataset of raw data, capl_demo_data, which contains 500 rows of participant data on the 60 variables that are required by the capl package. Users can load the demo dataset and start exploring.

**data**(capl_demo_data)

**Fig 1:**
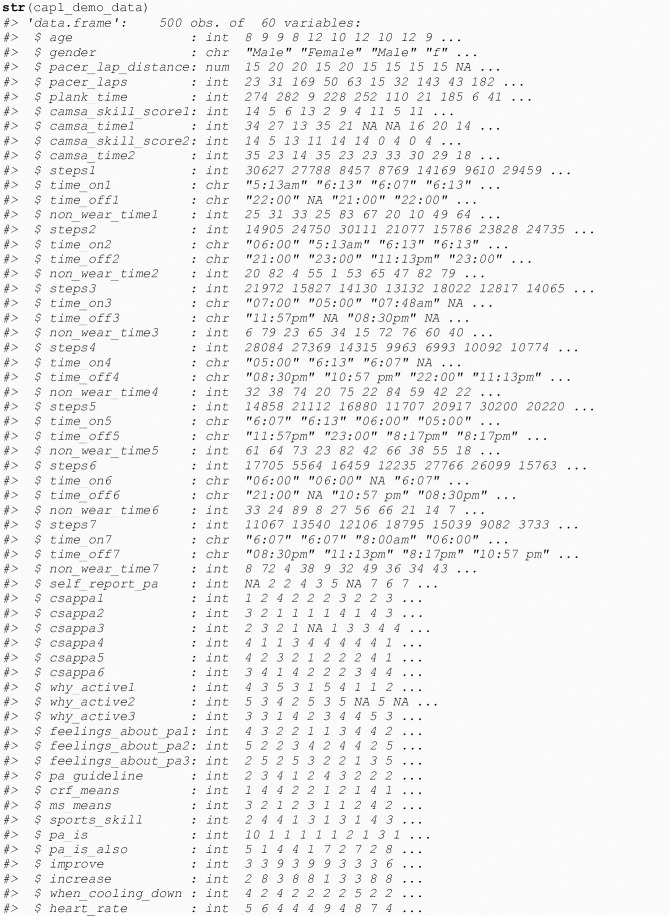
Structure of CAPL demo data.

The base R str() function allows users to get a sense of how the CAPL-2 raw data should be structured and named for upstream use in the capl package (see Fig 1).

The 60 required variables can also be quickly accessed by calling the base R colnames() function.

**colnames**(capl_demo_data)

*#> [1] "age"    "gender"    "pacer_lap_distance"*

*#> [4] "pacer_laps"    "plank_time"    "camsa_skill_score1"*

*#> [7] "camsa_time1"    "camsa_skill_score2"    "camsa_time2"*

*#> [10] "steps1"    "time_on1"    "time_off1"*

*#> [13] "non_wear_time1"    "steps2"    "time_on2"*

*#> [16] "time_off2"    "non_wear_time2"    "steps3"*

*#> [19] "time_on3"    "time_off3"    "non_wear_time3"*

*#> [22] "steps4"    "time_on4"    "time_off4"*

*#> [25] "non_wear_time4"    "steps5"    "time_on5"*

*#> [28] "time_off5"    "non_wear_time5"    "steps6"*

*#> [31] "time_on6"    "time_off6"    "non_wear_time6"*

*#> [34] "steps7"    "time_on7"    "time_off7"*

*#> [37] "non_wear_time7"    "self_report_pa"    "csappa1"*

*#> [40] "csappa2"    "csappa3"    "csappa4"*

*#> [43] "csappa5"    "csappa6"    "why_active1"*

*#> [46] "why_active2"    "why_active3"    "feelings_about_pa1"*

*#> [49] "feelings_about_pa2"    "feelings_about_pa3"    "pa_guideline"*

*#> [52] "crf_means"    "ms_means"    "sports_skill"*

*#> [55] "pa_is"    "pa_is_also"    "improve"*

*#> [58] "increase"    "when_cooling_down"    "heart_rate"*

### 2.5 Generating demo raw data

The capl package is also equipped with the get_capl_demo_data() function. This function allows users to randomly generate demo raw data and takes one parameter, n (set to 500 by default). This parameter is used to specify how many rows of demo raw data to generate and must, therefore, be an integer and greater than zero. Users, for example, can randomly generate demo raw data for 10,000 participants by executing a single line of code:

capl_demo_data2 <- **get_capl_demo_data**(n = 10000)

The base R str() function can be called to verify how many rows of data were created.

**str**(capl_demo_data2)

*#> ’data*.*frame’*:        *10000 obs*. *of 60 variables*:

*#> $ age*:        *int 10 10 9 12 9 12 10 11 9 11* …

*#> $ gender*:        *chr "f" "Boy" "g" "Female"* …

*#> $ pacer_lap_distance*:        *num 20 20 15 20 15 20 15 20 15 20* …

*#> $ pacer_laps*:        *int 75 93 102 131 96 151 129 150 127 10* …

*#> $ plank_time*:        *int 132 125 120 38 37 173 164 137 267 38* …

*#> $ camsa_skill_score1*:        *int 8 NA 7 5 6 6 10 4 10 7* …

*#> $ camsa_time1*:        *int 19 16 34 NA 32 16 19 20 25 25* …

*#> $ camsa_skill_score2*:        *int 7 10 9 6 10 8 6 6 7 11* …

*#> $ camsa_time2*:        *int 25 32 35 23 35 14 29 27 18 24* …

*#> $ steps1*:        *int 19261 22363 1181 5950 7020 21141 22435 18804 16575* …

*### For complete output*, *refer to the capl vignette*

### 2.6 Exporting data to Excel

If users prefer to examine the CAPL-2 demo raw data in a workbook, the export_capl_data() function allows them to export data objects to Excel.

**export_capl_data**(capl_demo_data2, "c:/users/joel/desktop/capl_demo_data2.xlsx")

### 2.7 Renaming variables

**Fig 2:**
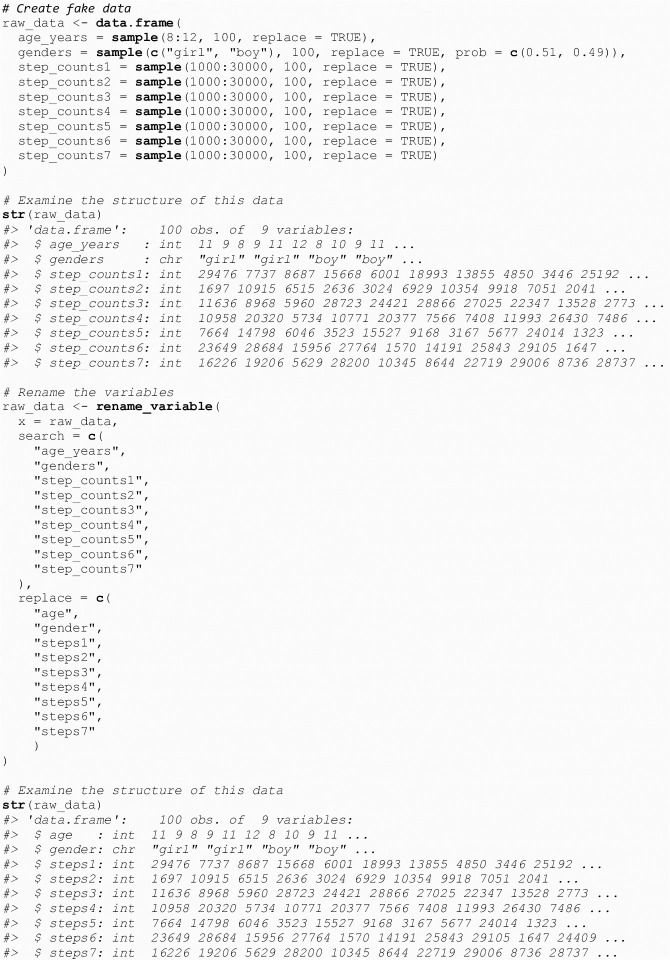
Rename variables.

If users import their own raw data and plan to use the main function (get_capl()) in the capl package to compute CAPL-2 scores and interpretations, they must ensure their variable names match the names of the 60 required variables. Users can rename their variables by calling the rename_variable() function (see Fig 2). This function takes three parameters: x, search, and replace. The x parameter must be the raw data object, the search parameter must be a character vector representing the variable name(s) to be renamed, and the replace parameter must be a character vector representing the new names for the variables specified in the search parameter. Below we show how to rename variables using a fake dataset called raw_data.

### 2.8 Eliminating noisy errors with validation

One of the coding philosophies behind the capl package is to create a “quiet” user experience by suppressing “noisy” error messages via validation. That is, the capl package returns missing or invalid values as NA values instead of throwing “noisy” errors that halt code execution. As important as error messages are, there is potential for many error messages to be thrown in the capl package due to the large number of computations performed across a diverse set of variables. This might discourage some users who are not able to eliminate these error messages in a timely manner. We have, therefore, opted to develop a package that offers a “quiet” user experience. If any variable is missing, for example, the get_capl() function will continue to execute without throwing error messages. The get_missing_capl_variables() function will create required variables that are missing and populate these variables with NA values. In order to implement the validation philosophy, every capl function enlists helper functions to validate the data. If a given value is not of the correct class or out of range, an NA will be returned.

#### 2.8.1 Validation functions in the capl package

There are eight validation functions included in the capl package (displayed in alphabetical order) to help provide a “quiet” user experience:

○ validate_age()

○ validate_character()

○ validate_domain_score()

○ validate_gender()

○ validate_integer()

○ validate_number()

○ validate_scale()

○ validate_steps()

Users can learn more about these functions by accessing the documentation within the R environment.

?validate_age

?validate_character

?validate_domain_score

?validate_gender

?validate_integer

?validate_number

?validate_scale

?validate_steps

Sections 2.7.2 and 2.7.3 illustrate examples of validation.

#### 2.8.2 Validation of age

The CAPL-2 is currently validated with 8- to 12-year-old children. However, when a function requires the age variable to execute a computation (e.g., get_capl_interpretation()), the age variable is validated via the validate_age() function.

validated_age <- **validate_age**(**c**(7, 8, 9, 10, 11, 12, 13, "", NA, "12", 8.5))

Notice the NA values in the results.

validated_age

*#> [1] NA 8 9 10 11 12 NA NA NA 12 8*

The first element is NA because the original value is 7 and the next five elements are identical to their original values because they are integers between 8 and 12. Recall that the CAPL-2 is validated with children aged 8 to 12 years, hence why the first value is NA (i.e., outside the validated range). The next two elements because the original values ("" and NA) are obviously invalid. The last element is 8, but notice that the original value is a decimal. Because 8.5 is between 8 and 12, it is considered valid but the floor of the value is returned since CAPL-2 performs age-specific computations based on integer age.

#### 2.8.3 Validation of gender

The CAPL-2 is currently validated for children who identify as boys or girls. When a function requires the gender variable to execute a computation, the gender variable is validated via the validate_gender() function.

validated_gender <- **validate_gender**(**c**("Girl", "GIRL", "g", "G", "Female", "f", "F", "", NA, 1))

validated_gender

*#> [1] "girl" "girl" "girl" "girl" "girl" "girl" "girl" NA      NA      "girl"*

Notice the results again. This function accepts a number of case-insensitive options (e.g., “Girl”, “G”, “female”, “F”, 1) for the female gender and returns a standardized “girl” value. The two elements that are returned as NA have original values that are obviously invalid ("" and NA). The validate_gender() function behaves in a similar fashion for the male gender; it also accepts a number of case-insensitive options and returns a standardized “boy” value.

validated_gender <- **validate_gender**(**c**("Boy", "BOY", "b", "B", "Male", "m", "M", "", NA, 0))

validated_gender

*#> [1] "boy" "boy" "boy" "boy" "boy" "boy" "boy" NA      NA      "boy"*

## 3. Computing CAPL-2 scores and interpretations

**Fig 3:**
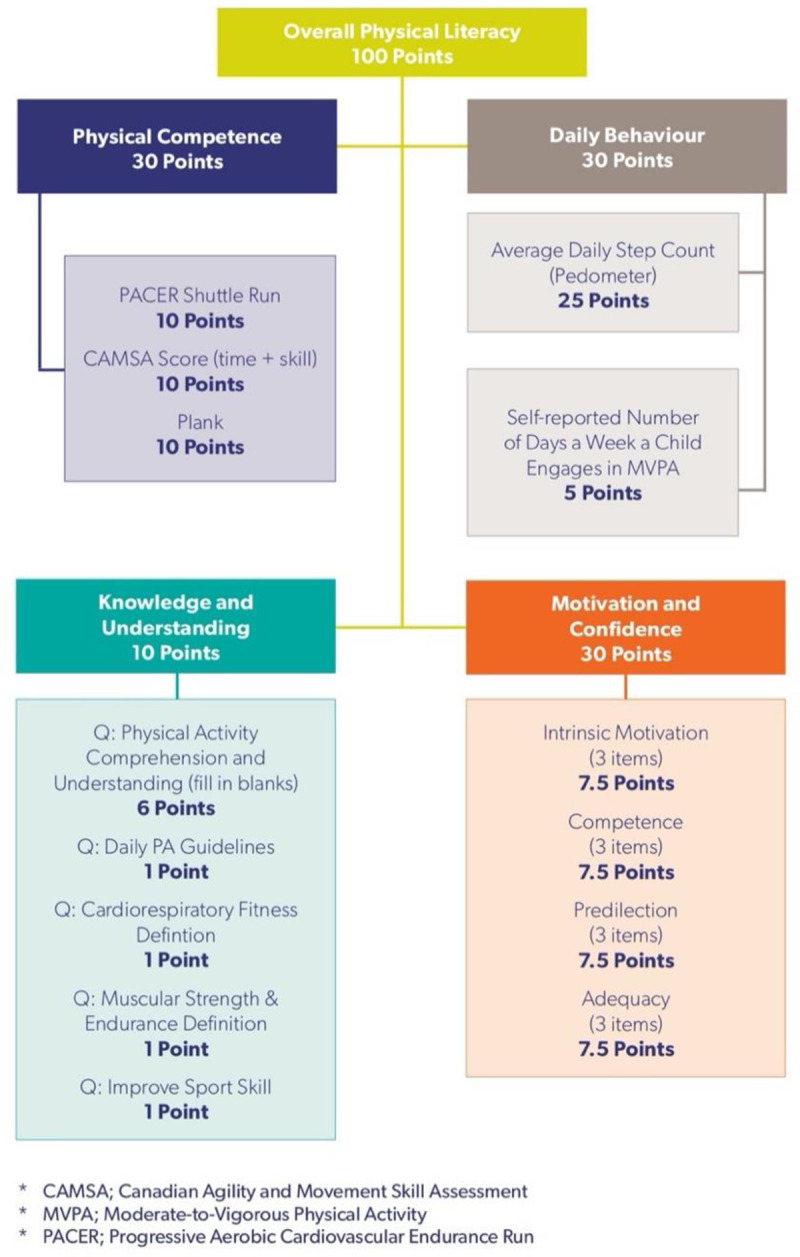
CAPL-2 scoring system. Reprinted from www.capl-eclp.ca/capl-manual under a CC BY license, with permission from Healthy Active Living and Obesity Research Group, original copyright 2017.

**Fig 4:**
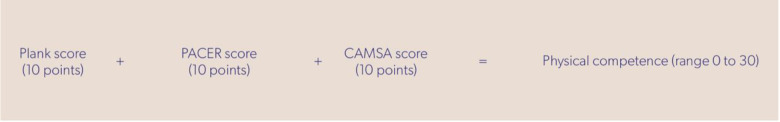
Formula for computing the physical competence score. Reprinted from www.capl-eclp.ca/capl-manual under a CC BY license, with permission from Healthy Active Living and Obesity Research Group, original copyright 2017.

The CAPL-2 scoring system is outlined in Fig 3 and in the CAPL-2 manual on page 7 (www.capl-eclp.ca/capl-manual):

The main function in the capl package is the get_capl() function. This function takes two parameters, raw_data and sort. It computes the CAPL-2 scores in Fig 4 above and their associated age- and gender-specific interpretations, row by row, by calling the other functions in the capl package. The raw_data parameter must be structured as a data frame and contain the raw data. The sort parameter is set to “asis” by default. This means the new computed variables will be added to the data frame as they are computed. If sort is set to “abc”, all variables will be sorted alphabetically whereas if sort is set to “zyx”, all variables will be sorted in reverse alphabetical order. Once the raw data has been imported, computing the CAPL-2 scores and interpretations is as simple as executing one line of code:

capl_results <- **get_capl**(raw_data = capl_demo_data, sort = "asis")

**Fig 5:**
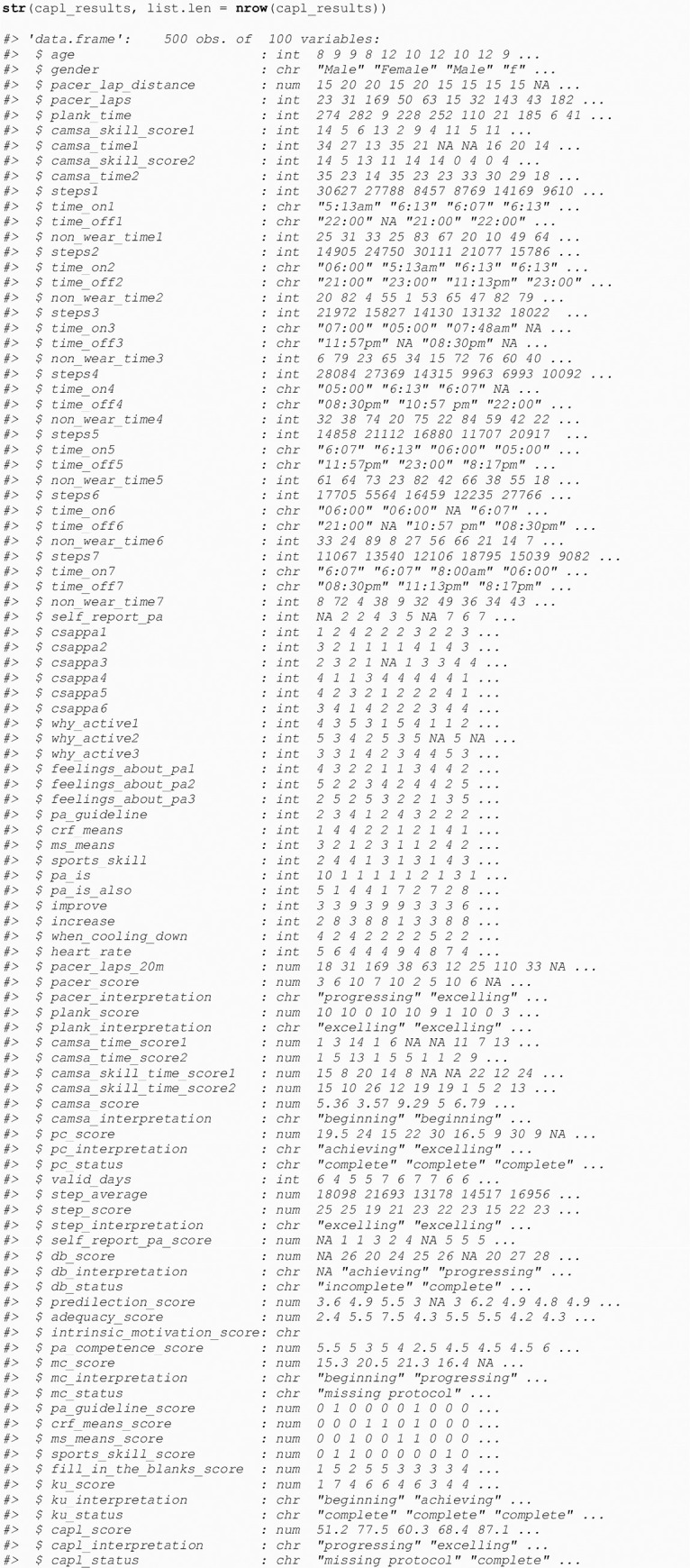
Confirmation of forty new computed variables.

The 40 new computed variables related to/including the CAPL-2 scores and interpretations can be confirmed by calling the base R str() function (see Fig 5). As illustrated on the first line of the output, there are now 500 rows of participant data on 100 variables.

### 3.1 Forty new variables computed by get_capl()

The 40 new variables related to/including the CAPL-2 scores and interpretations that are outputted from the get_capl() function include:

○ pacer_laps_20m

○ pacer_score

○ pacer_interpretation

○ plank_score

○ plank_interpretation

○ camsa_time_score1

○ camsa_time_score2

○ camsa_skill_time_score1

○ camsa_skill_time_score2

○ camsa_score

○ camsa_interpretation

○ pc_score

○ pc_interpretation

○ pc_status

○ step_average

○ valid_days

○ step_score

○ step_interpretation

○ self_report_pa_score

○ db_score

○ db_interpretation

○ db_status

○ predilection_score

○ adequacy_score

○ intrinsic_motivation_score

○ pa_competence_score

○ mc_score

○ mc_interpretation

○ mc_status

○ pa_guideline_score

○ crf_means_score

○ ms_means_score

○ sports_skill_score

○ fill_in_the_blanks_score

○ ku_score

○ ku_interpretation

○ ku_status

○ capl_score

○ capl_interpretation

○ capl_status

## 4.0 Computing CAPL-2 scores and interpretations manually

Some users may want to validate and compute individual variables and scores. The following sections introduce the helper functions in the order they appear when called in the get_capl() function.

### 4.1 Physical competence functions

As illustrated in Fig 4 and in the CAPL-2 manual on page 43 (www.capl-eclp.ca/capl-manual), the physical competence score is computed by summing the plank, PACER and CAMSA scores:

#### 4.1.1 PACER 20-metre laps

The pacer_laps_20m() function is used to convert PACER (Progressive Aerobic Cardiovascular Endurance Run) 15-metre shuttle run laps to 20-metre shuttle run laps. If laps are already 20-metre laps, the data are returned as is unless outside the valid range (1-229). This variable is used to compute the PACER score.

capl_demo_data$pacer_laps_20m <- **get_pacer_20m_laps**(

    lap_distance = capl_demo_data$pacer_lap_distance,

    laps_run = capl_demo_data$pacer_laps

)

capl_demo_data$pacer_laps_20m

*#> [1] 18 31 169 38 63 12 25 110 33 NA 127 62 39 19 NA 84 145 166*

*#> [19] 108 125 98 147 85 49 4 118 144 85 122 85 197 5 184 19 63 112*

*#> [37] 89 46 178 35 69 122 54 79 120 85 1 187 59 178 47 55 89 98*

*#> [55] 79 119 11 70 89 88 68 82 116 38 152 195 4 69 100 99 NA 88*

*#> [73] 57 43 98 125 127 5 16 173 20 33 89 99 39 35 43 100 177 15*

*#> [91] 141 141 39 NA 8 41 43 2 101 NA 54 78 90 176 40 2 122 58*

*#> [109] 98 5 51 112 101 122 12 177 38 92 31 53 102 200 138 166 62 31*

*#> [127] 86 75 87 56 147 140 45 51 136 74 148 194 67 142 172 121 NA 78*

*#> [145] 40 18 59 190 78 69 33 78 78 58 84 122 27 199 147 75 180 84*

*#> [163] 36 129 14 133 70 65 8 43 110 64 69 120 125 28 142 115 108 14*

*### For complete output*, *refer to the capl vignette*

#### 4.1.2 PACER score

The get_pacer_score() function computes a PACER score that ranges from zero to 10 based on the number of PACER laps run at a 20-metre distance. This score is used to compute the physical competence domain score variable.

capl_demo_data$pacer_score <- **get_pacer_score**(capl_demo_data$pacer_laps_20m)

capl_demo_data$pacer_score

*#> [1] 3 6 10 7 10 2 5 10 6 NA 10 10 7 3 NA 10 10 10 10 10 10 10 10 9 0*

*#> [26] 10 10 10 10 10 10 1 10 3 10 10 10 9 10 7 10 10 10 10 10 10 0 10 10 10*

*#> [51] 9 10 10 10 10 10 2 10 10 10 10 10 10 7 10 10 0 10 10 10 NA 10 10 8 10*

*#> [76] 10 10 1 3 10 4 6 10 10 7 7 8 10 10 3 10 10 7 NA 1 8 8 0 10 NA*

*#> [101] 10 10 10 10 8 0 10 10 10 1 10 10 10 10 2 10 7 10 6 10 10 10 10 10 10*

*#> [126] 6 10 10 10 10 10 10 9 10 10 10 10 10 10 10 10 10 NA 10 8 3 10 10 10 10*

*#> [151] 6 10 10 10 10 10 5 10 10 10 10 10 7 10 2 10 10 10 1 8 10 10 10 10 10*

*#> [176] 5 10 10 10 2 10 10 8 10 10 10 10 10 10 10 10 10 5 4 8 10 10 3 10 10*

*#> [201] 3 5 10 10 10 10 10 3 NA 10 10 6 7 10 10 NA 5 9 10 10 7 9 NA 10 9*

*#> [226] 10 10 9 10 10 NA 6 10 7 10 5 10 10 10 10 10 10 NA 10 10 10 1 10 NA 10*

*### For complete output*, *refer to the capl vignette*

#### 4.1.3 PACER interpretation

The get_capl_interpretation() function computes an age- and gender-specific CAPL-2 interpretation for a given CAPL-2 protocol or domain score.

capl_demo_data$pacer_interpretation <- **get_capl_interpretation**(

    age = capl_demo_data$age,

    gender = capl_demo_data$gender,

    score = capl_demo_data$pacer_score,

    protocol = "pacer"

)

capl_demo_data$pacer_interpretation

*#> [1] "beginning" "beginning" "progressing" "beginning" "beginning"*

*#> [6] "beginning" "beginning" "progressing" "beginning" NA*

*#> [11] NA "beginning" NA NA NA*

*#> [16] "progressing" "beginning" "progressing" "beginning" "progressing"*

*#> [21] "beginning" "beginning" "progressing" NA NA*

*#> [26] NA "beginning" NA "beginning" "progressing"*

*#> [31] "progressing" "beginning" "beginning" NA "beginning"*

*#> [36] NA NA "beginning" "progressing" "beginning"*

*#> [41] "beginning" "progressing" "progressing" "beginning" "beginning"*

*#> [46] "progressing" NA "progressing" NA NA*

*### For complete output*, *refer to the capl vignette*

#### 4.1.4 Plank score

The get_plank_score() function computes a plank score that ranges from zero to 10 based on the duration of time (in seconds) for which a plank is held. This score is used to compute the physical competence domain score.

capl_demo_data$plank_score <- **get_plank_score**(capl_demo_data$plank_time)

capl_demo_data$plank_score

*#> [1] 10 10 0 10 10 9 1 10 0 3 10 10 10 10 10 10 10 10 0 10 10 5 3 10 9*

*#> [26] 10 10 0 10 5 0 0 5 10 5 10 4 7 10 3 6 10 0 10 8 6 10 10 0 10*

*#> [51] 5 9 10 10 10 10 10 10 10 10 10 8 0 10 4 10 2 9 0 6 10 10 7 10 4*

*#> [76] 10 10 10 6 0 10 10 10 10 9 10 10 10 10 5 0 10 10 10 1 8 10 10 8 2*

*#> [101] 4 10 0 10 2 10 9 10 3 10 10 5 10 8 4 10 10 10 10 0 10 10 0 10 10*

*#> [126] 10 10 10 10 10 5 10 3 10 0 10 7 0 10 7 0 10 10 10 6 10 10 10 0 1*

*#> [151] 5 10 10 5 2 10 4 10 10 10 10 1 10 0 8 10 10 0 7 7 10 10 1 10 10*

*#> [176] 10 10 10 10 10 1 0 9 10 10 10 10 10 10 10 6 3 3 10 10 1 10 10 10 8*

*#> [201] 0 0 10 4 10 3 10 10 3 0 10 3 10 10 10 7 10 0 10 10 10 1 10 10 0*

*#> [226] 10 10 10 2 4 10 0 4 0 10 10 0 6 10 10 10 0 10 10 8 10 10 10 0 10*

*### For complete output*, *refer to the capl vignette*

#### 4.1.5 Plank interpretation

The get_capl_interpretation() function computes an age- and gender-specific CAPL-2 interpretation for a given CAPL-2 protocol or domain score.

capl_demo_data$plank_interpretation <- **get_capl_interpretation**(

    age = capl_demo_data$age,

    gender = capl_demo_data$gender,

    score = capl_demo_data$plank_time,

    protocol = "plank"

)

capl_demo_data$plank_interpretation

*#> [1] "excelling" "excelling" "beginning" "excelling" "excelling"*

*#> [6] "excelling" "beginning" "excelling" "beginning" "progressing"*

*#> [11] NA "excelling" NA NA "excelling"*

*#> [16] "excelling" "excelling" "excelling" "beginning" "excelling"*

*#> [21] "excelling" "achieving" "progressing" NA NA*

*#> [26] NA "excelling" NA "excelling" "progressing"*

*#> [31] "beginning" "beginning" "progressing" NA "progressing"*

*#> [36] NA NA "achieving" "excelling" "progressing"*

*#> [41] "progressing" "excelling" "beginning" "excelling" "achieving"*

*#> [46] "progressing" "excelling" "excelling" NA NA*

*### For complete output*, *refer to the capl vignette*

#### 4.1.6 CAMSA time score

**Fig 6:**
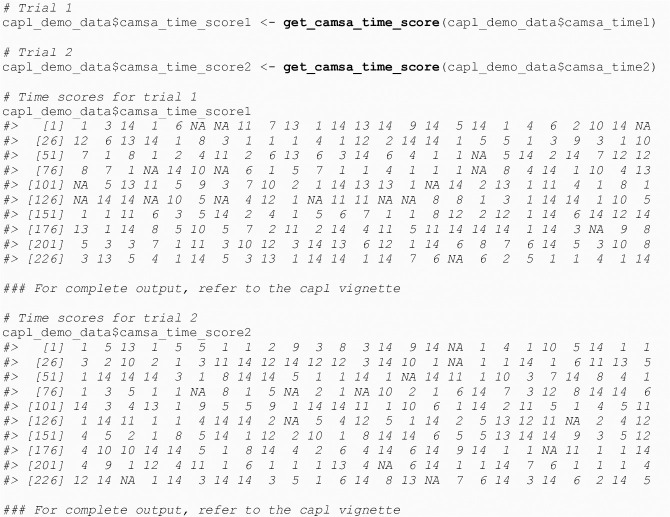
Calucate CAMSA time score.

The get_camsa_time_score() function computes the CAMSA (Canadian Agility and Movement Skill Assessment) time score that ranges from one to 14 based on the time taken (in seconds) to complete a trial (see Fig 6).

#### 4.1.7 CAMSA skill + time score

**Fig 7:**
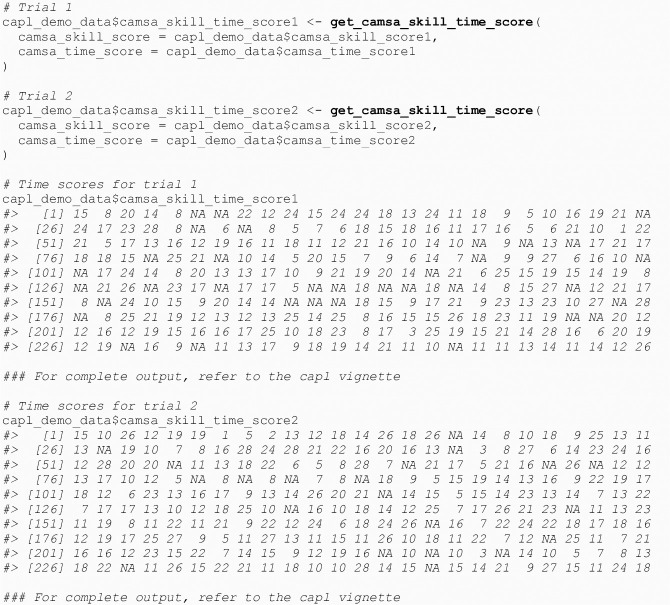
Calculate CAMSA skill + time score.

The get_camsa_skill_time_score() function computes the CAMSA skill + time score for a given trial that ranges from one to 28 (see Fig 7). This score is used to compute the CAMSA score.

#### 4.1.8 CAMSA score

The get_camsa_score() function computes the maximum CAMSA skill + time score for two trials and then divides by 2.8 so that the score is out of 10. This score is used to compute the physical literacy score.

capl_demo_data$camsa_score <- **get_camsa_score**(

    camsa_skill_time_score1 = capl_demo_data$camsa_skill_time_score1,

    camsa_skill_time_score2 = capl_demo_data$camsa_skill_time_score2

)

capl_demo_data$camsa_score

*#> [1] 5*.*357143 3*.*571429 9*.*285714 5*.*000000 6*.*785714 NA NA*

*#> [8] 7*.*857143 4*.*285714 8*.*571429 5*.*357143 8*.*571429 8*.*571429 9*.*285714*

*#> [15] 6*.*428571 9*.*285714 NA 6*.*428571 3*.*214286 3*.*571429 6*.*428571*

*#> [22] 5*.*714286 8*.*928571 7*.*500000 NA 8*.*571429 NA 8*.*214286*

*#> [29] 10*.*000000 2*.*857143 NA 5*.*714286 NA 8*.*571429 10*.*000000*

*#> [36] 7*.*500000 7*.*857143 6*.*428571 7*.*142857 6*.*428571 5*.*714286 NA*

*#> [43] 6*.*071429 5*.*714286 9*.*642857 2*.*142857 7*.*500000 8*.*214286 8*.*571429*

*#> [50] 7*.*857143 7*.*500000 10*.*000000 7*.*142857 7*.*142857 NA 4*.*285714*

*#> [57] 6*.*785714 6*.*428571 7*.*857143 6*.*428571 3*.*928571 4*.*285714 10*.*000000*

*#> [64] 5*.*714286 NA 7*.*500000 6*.*071429 NA 7*.*500000 NA*

*### For complete output*, *refer to the capl vignette*

#### 4.1.9 CAMSA interpretation

The get_capl_interpretation() function computes an age- and gender-specific CAPL-2 interpretation for a given CAPL-2 protocol or domain score

capl_demo_data$camsa_interpretation <- **get_capl_interpretation**(

    age = capl_demo_data$age,

    gender = capl_demo_data$gender,

    score = capl_demo_data$camsa_score,

    protocol = "camsa"

)

capl_demo_data$camsa_interpretation

*#> [1] "beginning" "beginning" "excelling" "beginning" "progressing"*

*#> [6] NA NA "progressing" "beginning" "excelling"*

*#> [11] NA "achieving" NA NA "progressing"*

*#> [16] "excelling" NA "progressing" "beginning" "beginning"*

*#> [21] "progressing" "beginning" "excelling" NA NA*

*#> [26] NA NA NA "excelling" "beginning"*

*#> [31] NA "beginning" NA NA "excelling"*

*#> [36] NA NA "progressing" "progressing" "progressing"*

*#> [41] "beginning" NA "progressing" "beginning" "excelling"*

*#> [46] "beginning" "progressing" "achieving" NA NA*

*### For complete output*, *refer to the capl vignette*

#### 4.1.10 Physical competence score

The get_pc_score() function computes a physical competence domain score that ranges from zero to 30 based on the PACER, plank and CAMSA scores. If one protocol score is missing or invalid, a weighted domain score is computed from the other two protocol scores. This score is used to compute the physical competence domain score.

capl_demo_data$pc_score <- **get_pc_score**(

    pacer_score = capl_demo_data$pacer_score,

    plank_score = capl_demo_data$plank_score,

    camsa_score = capl_demo_data$camsa_score

)

capl_demo_data$pc_score

*#> [1] 19*.*5 24*.*0 15*.*0 22*.*0 30*.*0 16*.*5 9*.*0 30*.*0 9*.*0 NA 30*.*0 30*.*0 25*.*5 19*.*5 NA*

*#> [16] 30*.*0 30*.*0 30*.*0 15*.*0 30*.*0 30*.*0 22*.*5 19*.*5 28*.*5 13*.*5 30*.*0 30*.*0 15*.*0 30*.*0 22*.*5*

*#> [31] 15*.*0 1*.*5 22*.*5 19*.*5 25*.*0 30*.*0 21*.*0 24*.*0 30*.*0 15*.*0 24*.*0 30*.*0 15*.*0 30*.*0 27*.*0*

*#> [46] 24*.*0 15*.*0 30*.*0 15*.*0 30*.*0 21*.*0 29*.*0 30*.*0 30*.*0 30*.*0 30*.*0 18*.*0 30*.*0 30*.*0 30*.*0*

*#> [61] 30*.*0 27*.*0 20*.*0 25*.*5 21*.*0 30*.*0 3*.*0 28*.*5 15*.*0 24*.*0 NA 30*.*0 25*.*5 27*.*0 21*.*0*

*#> [76] 30*.*0 30*.*0 16*.*5 13*.*5 15*.*0 21*.*0 24*.*0 30*.*0 25*.*0 24*.*0 25*.*5 27*.*0 30*.*0 30*.*0 12*.*0*

*#> [91] 15*.*0 30*.*0 25*.*5 22*.*5 3*.*0 24*.*0 27*.*0 15*.*0 27*.*0 NA 21*.*0 30*.*0 15*.*0 30*.*0 15*.*0*

*#> [106] 15*.*0 28*.*5 30*.*0 19*.*5 16*.*0 30*.*0 22*.*5 30*.*0 27*.*0 11*.*0 30*.*0 25*.*5 30*.*0 24*.*0 15*.*0*

*#> [121] 30*.*0 30*.*0 15*.*0 30*.*0 30*.*0 24*.*0 30*.*0 30*.*0 30*.*0 30*.*0 22*.*5 30*.*0 18*.*0 30*.*0 15*.*0*

*#> [136] 30*.*0 25*.*5 15*.*0 30*.*0 25*.*5 15*.*0 30*.*0 NA 30*.*0 21*.*0 19*.*5 30*.*0 30*.*0 15*.*0 16*.*5*

*### For complete output*, *refer to the capl vignette*

#### 4.1.11 Physical competence interpretation

The get_capl_interpretation() function computes an age- and gender-specific CAPL-2 interpretation for a given CAPL-2 protocol or domain score.

capl_demo_data$pc_interpretation <- **get_capl_interpretation**(

    age = capl_demo_data$age,

    gender = capl_demo_data$gender,

    score = capl_demo_data$pc_score,

    protocol = "pc"

)

capl_demo_data$pc_interpretation

*#> [1] "achieving" "excelling" "progressing" "excelling" "excelling"*

*#> [6] "progressing" "beginning" "excelling" "beginning" NA*

*#> [11] NA            "excelling" NA            NA            NA*

*#> [16] "excelling" "excelling" "excelling" "progressing" "excelling"*

*#> [21] "excelling" "excelling" "achieving" NA            NA*

*#> [26] NA            "excelling" NA            "excelling" "achieving"*

*#> [31] "progressing" "beginning" "excelling" NA            "excelling"*

*#> [36] NA            NA             "excelling" "excelling" "progressing"*

*#> [41] "excelling" "excelling" "progressing" "excelling" "excelling"*

*#> [46] "excelling" "progressing" "excelling" NA            NA*

*### For complete output*, *refer to the capl vignette*

#### 4.1.12 Physical competence domain status

The get_capl_domain_status() function computes the status (“complete”, “missing interpretation”, “missing protocol” or “incomplete”) of a CAPL domain.

capl_demo_data$pc_status <- **get_capl_domain_status**(

    x = capl_demo_data,

    domain = "pc"

)

capl_demo_data$pc_status

*#> [1] "complete"            "complete"            "complete"*

*#> [4] "complete"            "complete"            "missing protocol"*

*#> [7] "missing protocol"            "complete"            "complete"*

*#> [10] "incomplete"            "missing interpretation"    "complete"*

*#> [13] "missing interpretation"     "missing interpretation"     "incomplete"*

*#> [16] "complete"            "missing protocol"            "complete"*

*#> [19] "complete"            "complete"            "complete"*

*#> [22] "complete"            "complete"            "missing interpretation"*

*#> [25] "missing interpretation"    "missing interpretation"     "missing protocol"*

*#> [28] "missing interpretation"      "complete"            "complete"*

*### For complete output*, *refer to the capl vignette*

### 4.2 Daily behaviour

**Fig 8:**
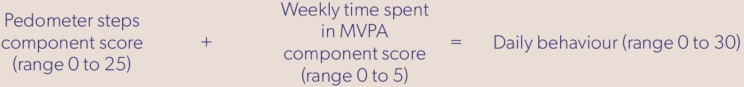
Formula for computing the daily behaviour score. Reprinted from www.capl-eclp.ca/capl-manual under a CC BY license, with permission from Healthy Active Living and Obesity Research Group, original copyright 2017.

As illustrated in Fig 8 and in the CAPL-2 manual on page 26 (www.capl-eclp.ca/capl-manual), the formula for computing the daily behaviour score is:

#### 4.2.1 Step average

The get_step_average() function computes the daily arithmetic mean of a week of steps measured by pedometry. This variable is used to compute the step score.

step_df <- **get_step_average**(

    capl_demo_data

)

**Fig 9:**
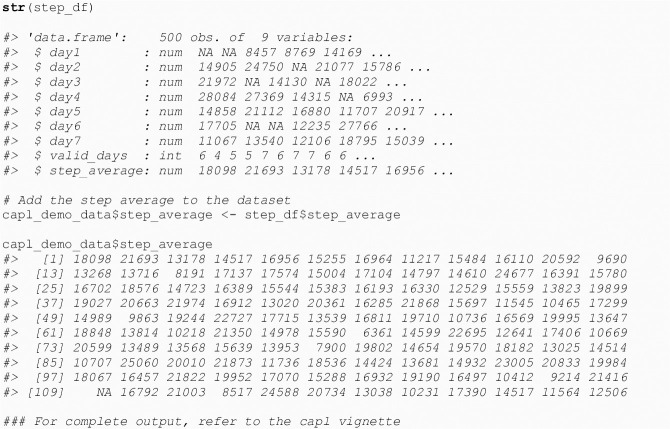
Calculate daily step average.

The get_step_average() function returns a data frame with nine columns: steps1 (validated), steps2 (validated), steps3 (validated), steps4 (validated), steps5 (validated), steps6 (validated), steps7 (validated), valid_days and step_average (see Fig 9).

**Fig 10:**
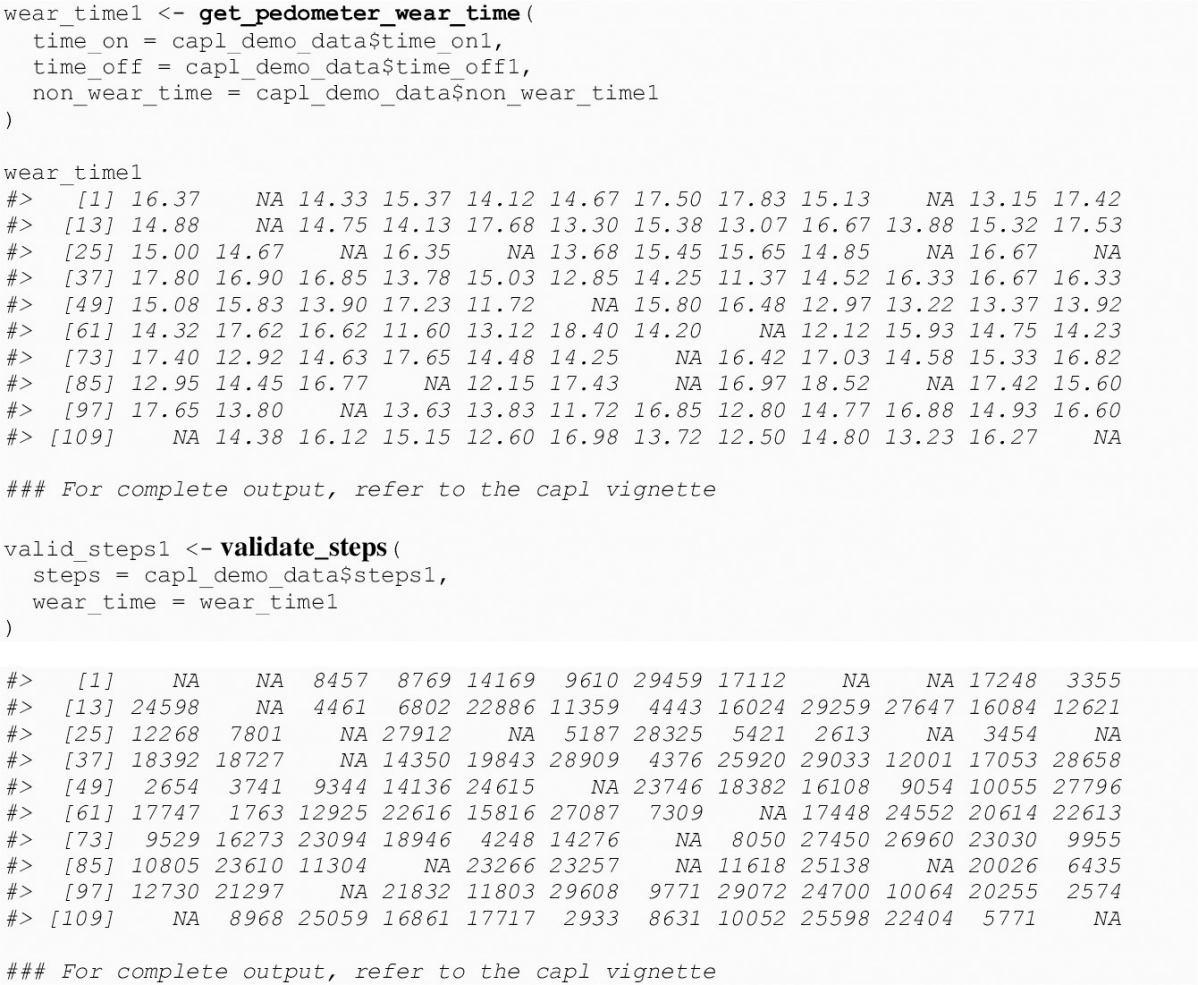
Functions called by the get_step_average() function.

There must be at least four valid days of pedometer step counts for an arithmetic mean to be computed. If there are less than four valid days, one of the step values from a valid day is randomly sampled and used for the fourth valid day before computing the mean. Other important capl functions called by the get_step_average() function include get_pedometer_wear_time() and validate_steps() (see Fig 10).

#### 4.2.2 Step score

The get_step_score() function computes a step score that ranges from 0 to 25 based on the average daily steps taken as measured by a pedometer. This score is used to compute the daily behaviour domain score.

capl_demo_data$step_score <- **get_step_score**(capl_demo_data$step_average)

capl_demo_data$step_score

*#> [1] 25 25 19 21 23 22 23 15 22 23 25 12 19 20 9 24 24 22 24 21 21 25 23 22 23*

*#> [26] 25 21 23 22 22 23 23 18 22 20 25 25 25 25 23 19 25 23 25 22 16 13 24 21 12*

*#> [51] 25 25 24 20 23 25 14 23 25 20 25 20 13 25 21 22 5 21 25 18 24 14 25 19 20*

*#> [76] 22 20 8 25 21 25 25 19 21 14 25 25 25 16 25 21 20 21 25 25 25 25 23 25 25*

*#> [101] 24 22 23 25 23 13 11 25 NA 23 25 10 25 25 19 13 24 21 16 18 25 25 24 20 19*

*#> [126] 22 11 23 14 15 17 24 24 14 23 25 21 20 22 13 18 25 20 18 19 12 14 25 23 25*

*#> [151] 22 17 22 18 22 23 25 23 14 19 18 25 25 17 25 16 25 22 19 22 22 6 22 16 25*

*#> [176] 24 22 23 22 24 15 21 25 25 22 25 24 25 22 22 23 14 22 14 22 22 24 19 23 16*

*#> [201] 14 17 4 24 22 25 23 20 25 19 24 22 25 24 15 25 22 21 25 22 16 23 16 25 25*

*#> [226] 25 19 13 20 25 20 25 21 25 25 22 21 18 18 24 21 15 23 18 21 25 24 25 25 25*

*### For complete output*, *refer to the capl vignette*

#### 4.2.3 Self-reported physical activity score

The get_self_report_pa() function computes a score that ranges from zero to five based on the response to “During the past week (7 days), on how many days were you physically active for a total of at least 60 minutes per day? (all the time you spent in activities that increased your heart rate and made you breathe hard)?” in the CAPL-2 Questionnaire (www.capl-eclp.ca/wp-content/uploads/2018/02/CAPL-2-questionnaire.pdf). This score is used to compute the daily behaviour domain score.

capl_demo_data$self_report_pa_score <- **get_self_report_pa_score**(capl_demo_data$self_report_pa)

capl_demo_data$self_report_pa_score

*#> [1] NA 1 1 3 2 4 NA 5 5 5 NA 3 1 4 5 5 0 5 5 5 5 5 5 NA 5*

*#> [26] 3 4 5 4 1 2 0 5 5 5 5 4 3 4 2 4 5 5 5 2 5 5 NA 5 5*

*#> [51] 1 5 3 1 0 5 3 2 5 1 2 0 5 NA 3 5 4 0 3 NA 5 4 1 NA 0*

*#> [76] 5 0 2 0 NA 5 5 1 1 3 3 5 2 5 4 5 5 4 3 1 4 2 1 5 5*

*#> [101] 5 2 1 4 3 2 5 5 3 2 2 5 5 5 2 NA 5 1 5 5 NA 5 5 1 0*

*#> [126] 4 5 4 0 0 5 4 5 0 5 5 5 5 2 5 5 5 2 5 1 3 5 5 NA 5*

*#> [151] 5 0 2 1 0 5 3 3 2 0 5 1 5 4 0 3 5 5 3 5 5 5 0 5 4*

*#> [176] NA 5 5 4 5 3 4 3 0 2 1 5 3 0 5 1 5 5 5 5 0 2 3 0 1*

*#> [201] 0 1 5 5 5 2 5 3 4 2 5 5 5 5 2 5 5 1 0 1 5 4 1 5 3*

*#> [226] 4 3 5 0 5 2 5 0 NA 5 3 3 NA 2 0 4 5 5 5 5 4 0 3 3 2*

*### For complete output*, *refer to the capl vignette*

#### 4.2.4 Daily behaviour score

The get_db_score() function computes a daily behaviour domain score that ranges from 0 to 30 based on the step and self-reported physical activity scores. This score is used to compute the overall physical literacy score.

capl_demo_data$db_score <- **get_db_score**(

    step_score = capl_demo_data$step_score,

    self_report_pa_score = capl_demo_data$self_report_pa_score

)

capl_demo_data$db_score

*#> [1] NA 26 20 24 25 26 NA 20 27 28 NA 15 20 24 14 29 24 27 29 26 26 30 28 NA 28*

*#> [26] 28 25 28 26 23 25 23 23 27 25 30 29 28 29 25 23 30 28 30 24 21 18 NA 26 17*

*#> [51] 26 30 27 21 23 30 17 25 30 21 27 20 18 NA 24 27 9 21 28 NA 29 18 26 NA 20*

*#> [76] 27 20 10 25 NA 30 30 20 22 17 28 30 27 21 29 26 25 25 28 26 29 27 24 30 30*

*#> [101] 29 24 24 29 26 15 16 30 NA 25 27 15 30 30 21 NA 29 22 21 23 NA 30 29 21 19*

*#> [126] 26 16 27 14 15 22 28 29 14 28 30 26 25 24 18 23 30 22 23 20 15 19 30 NA 30*

*#> [151] 27 17 24 19 22 28 28 26 16 19 23 26 30 21 25 19 30 27 22 27 27 11 22 21 29*

*#> [176] NA 27 28 26 29 18 25 28 25 24 26 29 28 22 27 24 19 27 19 27 22 26 22 23 17*

*#> [201] 14 18 9 29 27 27 28 23 29 21 29 27 30 29 17 30 27 22 25 23 21 27 17 30 28*

*#> [226] 29 22 18 20 30 22 30 21 NA 30 25 24 NA 20 24 25 20 28 23 26 29 24 28 28 27*

*### For complete output*, *refer to the capl vignette*

#### 4.2.5 Daily behaviour interpretation

The get_capl_interpretation() function computes an age- and gender-specific CAPL-2 interpretation for a given CAPL-2 protocol or domain score.

capl_demo_data$db_interpretation <- **get_capl_interpretation**(

    age = capl_demo_data$age,

    gender = capl_demo_data$gender,

    score = capl_demo_data$db_score,

    protocol = "db"

)

capl_demo_data$db_interpretation

*#> [1] NA        "achieving" "progressing" "achieving" "achieving"*

*#> [6] "achieving" NA    "progressing" "excelling" "excelling"*

*#> [11] NA    "progressing" NA    NA    "progressing"*

*#> [16] "excelling" "achieving" "excelling" "excelling" "excelling"*

*#> [21] "achieving" "excelling" "excelling" NA    NA*

*#> [26] NA    "excelling" NA    "excelling" "achieving"*

*#> [31] "achieving" "achieving" "achieving" NA    "excelling"*

*#> [36] NA    NA    "excelling" "excelling" "achieving"*

*#> [41] "achieving" "excelling" "excelling" "excelling" "achieving"*

*#> [46] "progressing" "progressing" NA    NA    NA*

*### For complete output*, *refer to the capl vignette*

#### 4.2.6 Daily behaviour domain status

The get_capl_domain_status() function computes the status (“complete”, “missing interpretation”, “missing protocol”, or “incomplete”) of a CAPL domain.

capl_demo_data$db_status <- **get_capl_domain_status**(

    x = capl_demo_data,

    domain = "db"

)

capl_demo_data$db_status

*#> [1] "incomplete"        "complete"        "complete"*

*#> [4] "complete"        "complete"        "complete"*

*#> [7] "incomplete"        "complete"        "complete"*

*#> [10] "complete"        "incomplete"        "complete"*

*#> [13] "missing interpretation" "missing interpretation" "complete"*

*#> [16] "complete"        "complete"        "complete"*

*#> [19] "complete"        "complete"        "complete"*

*#> [22] "complete"        "complete"        "incomplete"*

*#> [25] "missing interpretation" "missing interpretation" "complete"*

*#> [28] "missing interpretation" "complete"        "complete"*

*### For complete output*, *refer to the capl vignette*

### 4.3 Motivation and confidence functions

**Fig 11:**
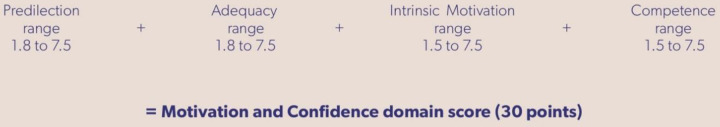
Formula for computing motivation and confidence score. Reprinted from www.capl-eclp.ca/capl-manual under a CC BY license, with permission from Healthy Active Living and Obesity Research Group, original copyright 2017.

As illustrated in Fig 11 and in the CAPL-2 manual on page 79 (www.capl-eclp.ca/capl-manual), the formula for computing the motivation and confidence score is:

#### 4.3.1 Predilection score

The get_predilection_score() function computes a predilection score that ranges from 1.8 to 7.5 based on responses to three items from the Children’s Self-Perception of Adequacy in and Predilection for Physical Activity as they appear in the CAPL-2 Questionnaire (www.capl-eclp.ca/wp-content/uploads/2018/02/CAPL-2-questionnaire.pdf). This score is used to compute the motivation and confidence domain score.

capl_demo_data$predilection_score <- **get_predilection_score**(

    csappa1 = capl_demo_data$csappa1,

    csappa3 = capl_demo_data$csappa3,

    csappa5 = capl_demo_data$csappa5

)

capl_demo_data$predilection_score

*#> [1] 3*.*6 4*.*9 5*.*5 3*.*0 NA 3*.*0 6*.*2 4*.*9 4*.*8 4*.*9 3*.*0 3*.*0 5*.*6 4*.*9 5*.*5 5*.*5 4*.*9 4*.*3*

*#> [19] 5*.*6 3*.*6 4*.*2 3*.*6 6*.*1 3*.*6 4*.*3 4*.*8 5*.*6 3*.*0 4*.*3 4*.*3 6*.*1 4*.*9 6*.*2 3*.*6 6*.*8 5*.*5*

*#> [37] 5*.*5 6*.*2 3*.*0 2*.*4 4*.*3 3*.*6 5*.*5 6*.*1 NA 4*.*3 2*.*4 4*.*9 4*.*9 NA 5*.*5 4*.*3 4*.*2 4*.*2*

*#> [55] 4*.*9 6*.*2 4*.*3 5*.*5 1*.*8 3*.*7 4*.*9 3*.*7 6*.*8 4*.*8 6*.*2 4*.*2 3*.*7 1*.*8 5*.*5 3*.*0 5*.*5 4*.*2*

*#> [73] 4*.*8 5*.*5 4*.*9 3*.*6 4*.*9 3*.*7 3*.*6 3*.*6 4*.*9 6*.*2 4*.*2 3*.*7 3*.*7 3*.*6 3*.*0 2*.*4 4*.*2 4*.*2*

*#> [91] 3*.*0 5*.*4 5*.*5 6*.*2 4*.*3 3*.*6 5*.*6 6*.*1 4*.*9 NA 4*.*2 5*.*5 6*.*8 4*.*8 4*.*9 4*.*9 4*.*9 2*.*4*

*#> [109] 4*.*8 3*.*0 3*.*6 3*.*6 4*.*9 3*.*6 4*.*3 3*.*7 2*.*4 3*.*0 3*.*6 4*.*2 4*.*3 4*.*3 6*.*8 5*.*6 4*.*9 6*.*2*

*#> [127] 3*.*0 3*.*0 5*.*5 3*.*6 4*.*2 3*.*7 6*.*1 3*.*7 3*.*6 6*.*2 6*.*1 4*.*8 3*.*6 4*.*9 1*.*8 5*.*6 3*.*0 4*.*9*

*#> [145] 4*.*9 4*.*8 3*.*0 4*.*8 4*.*2 4*.*9 2*.*4 5*.*5 3*.*7 4*.*3 3*.*6 3*.*0 4*.*8 5*.*5 7*.*5 4*.*9 4*.*8 4*.*2*

*#> [163] 3*.*0 2*.*4 5*.*5 2*.*4 5*.*5 6*.*1 3*.*6 5*.*5 4*.*3 4*.*2 4*.*3 NA 6*.*1 3*.*6 4*.*9 5*.*6 3*.*7 2*.*4*

*### For complete output*, *refer to the capl vignette*

#### 4.3.2 Adequacy score

The get_adequacy_score() function computes an adequacy score that ranges from 1.8 to 7.5 based on responses to three items from the Children’s Self-Perception of Adequacy in and Predilection for Physical Activity as they appear in the CAPL-2 Questionnaire (www.capl-eclp.ca/wp-content/uploads/2018/02/CAPL-2-questionnaire.pdf). This score is used to compute the motivation and confidence domain score.

capl_demo_data$adequacy_score <- **get_adequacy_score**(

    csappa2 = capl_demo_data$csappa2,

    csappa4 = capl_demo_data$csappa4,

    csappa6 = capl_demo_data$csappa6

)

capl_demo_data$adequacy_score

*#> [1] 2*.*4 5*.*5 7*.*5 4*.*3 5*.*5 5*.*5 4*.*2 4*.*3 3*.*6 4*.*3 6*.*2 4*.*9 4*.*9 4*.*2 4*.*3 5*.*6 4*.*2 4*.*8*

*#> [19] 4*.*8 6*.*2 4*.*3 4*.*8 4*.*9 3*.*6 6*.*8 4*.*3 6*.*2 6*.*2 4*.*3 7*.*5 6*.*1 5*.*6 4*.*9 6*.*2 6*.*2 2*.*4*

*#> [37] 4*.*2 5*.*6 5*.*6 4*.*2 5*.*5 4*.*9 5*.*5 4*.*9 4*.*2 6*.*2 5*.*6 6*.*2 4*.*9 4*.*2 3*.*6 3*.*7 3*.*6 6*.*8*

*#> [55] 1*.*8 3*.*6 5*.*6 4*.*9 3*.*6 3*.*0 3*.*6 4*.*9 3*.*7 4*.*9 3*.*6 3*.*7 4*.*2 5*.*6 6*.*1 4*.*9 3*.*6 3*.*0*

*#> [73] 2*.*4 4*.*3 6*.*1 3*.*0 3*.*0 3*.*7 4*.*9 4*.*3 5*.*4 5*.*5 3*.*0 2*.*4 6*.*1 5*.*6 5*.*5 3*.*7 4*.*2 2*.*4*

*#> [91] 5*.*6 4*.*9 3*.*0 5*.*6 4*.*9 3*.*6 4*.*9 6*.*8 4*.*9 2*.*4 5*.*6 4*.*8 4*.*2 3*.*0 4*.*9 3*.*0 3*.*0 3*.*7*

*#> [109] 4*.*9 3*.*7 6*.*2 4*.*9 5*.*5 5*.*6 5*.*6 4*.*8 3*.*7 6*.*2 3*.*0 5*.*4 4*.*9 3*.*6 5*.*4 3*.*0 4*.*8 3*.*6*

*#> [127] 4*.*8 4*.*2 4*.*2 2*.*4 4*.*9 4*.*3 6*.*2 2*.*4 4*.*9 4*.*9 4*.*9 4*.*2 4*.*9 5*.*5 4*.*9 5*.*5 7*.*5 5*.*6*

*#> [145] 5*.*5 3*.*0 4*.*9 6*.*1 4*.*9 5*.*5 3*.*0 5*.*6 6*.*2 4*.*9 4*.*3 4*.*9 5*.*6 4*.*2 2*.*4 5*.*5 4*.*9 3*.*7*

*#> [163] 4*.*3 4*.*3 4*.*9 4*.*3 6*.*1 4*.*8 4*.*9 5*.*5 6*.*2 6*.*1 3*.*6 2*.*4 3*.*6 4*.*9 6*.*2 4*.*3 4*.*3 4*.*2*

*### For complete output*, *refer to the capl vignette*

#### 4.3.3 Intrinsic motivation score

The get_intrinsic_motivation_score() function computes an intrinsic motivation score that ranges from 1.5 to 7.5 based on responses to three items from the Behavioral Regulation in Exercise Questionnaire (BREQ) as they appear in the CAPL-2 Questionnaire (www.capl-eclp.ca/wp-content/uploads/2018/02/CAPL-2-questionnaire.pdf). This score is used to compute the motivation and confidence domain score.

capl_demo_data$intrinsic_motivation_score <- **get_intrinsic_motivation_score**(

    why_active1 = capl_demo_data$why_active1,

    why_active2 = capl_demo_data$why_active2,

    why_active3 = capl_demo_data$why_active3

)

capl_demo_data$intrinsic_motivation_score

*#> [1] 6*.*0 4*.*5 5*.*0 4*.*5 4*.*0 5*.*5 6*.*5 NA 5*.*5 NA 6*.*5 5*.*0 4*.*0 3*.*5 NA 6*.*0 4*.*5 4*.*5*

*#> [19] 5*.*0 3*.*0 2*.*0 2*.*5 3*.*5 NA 5*.*0 4*.*0 4*.*5 7*.*0 3*.*5 4*.*0 NA 6*.*0 5*.*0 3*.*5 NA 4*.*5*

*#> [37] 4*.*5 NA 5*.*0 5*.*0 4*.*0 3*.*5 6*.*5 4*.*0 NA 4*.*5 3*.*0 NA 5*.*0 5*.*0 4*.*0 6*.*5 NA 4*.*0*

*#> [55] 5*.*0 3*.*5 6*.*5 2*.*5 6*.*5 5*.*0 6*.*0 5*.*5 4*.*5 3*.*5 3*.*0 2*.*5 4*.*5 4*.*5 4*.*5 4*.*5 4*.*5 3*.*0*

*#> [73] 4*.*5 4*.*5 5*.*0 5*.*0 2*.*5 6*.*5 5*.*5 4*.*5 6*.*0 2*.*5 4*.*0 7*.*0 4*.*5 NA 3*.*5 NA 3*.*5 3*.*0*

*#> [91] 4*.*0 4*.*0 5*.*0 5*.*0 2*.*5 NA 3*.*5 6*.*0 1*.*5 6*.*5 5*.*5 5*.*5 5*.*5 4*.*0 4*.*0 4*.*0 NA 3*.*0*

*#> [109] NA 4*.*0 5*.*0 5*.*5 3*.*5 4*.*0 3*.*0 4*.*0 6*.*5 3*.*5 6*.*0 3*.*0 4*.*5 6*.*0 NA 3*.*0 3*.*5 NA*

*#> [127] 5*.*0 3*.*0 NA 5*.*5 5*.*5 6*.*5 4*.*0 NA 5*.*0 5*.*0 5*.*0 NA 3*.*0 2*.*5 NA 4*.*0 6*.*0 5*.*0*

*#> [145] 5*.*0 3*.*5 4*.*5 5*.*0 NA 4*.*5 2*.*5 4*.*0 4*.*0 5*.*5 3*.*0 7*.*0 5*.*0 5*.*0 5*.*0 NA 5*.*5 7*.*5*

*#> [163] 4*.*0 2*.*5 3*.*5 5*.*5 NA 4*.*5 6*.*0 5*.*0 5*.*0 5*.*5 2*.*5 4*.*5 5*.*5 2*.*0 5*.*0 7*.*0 NA 6*.*0*

*### For complete output*, *refer to the capl vignette*

#### 4.3.4 Physical activity competence score

The get_pa_competence_score() function computes a physical activity competence score that ranges from 1.5 to 7.5 based on responses to three items from the Behavioural Regulation in Exercise Questionnaire as they appear in the CAPL-2 Questionnaire (www.capl-eclp.ca/wp-content/uploads/2018/02/CAPL-2-questionnaire.pdf). This score is used to compute the motivation and confidence domain score.

capl_demo_data$pa_competence_score <- **get_pa_competence_score**(

    feelings_about_pa1 = capl_demo_data$feelings_about_pa1,

    feelings_about_pa2 = capl_demo_data$feelings_about_pa2,

    feelings_about_pa3 = capl_demo_data$feelings_about_pa3

)

capl_demo_data$pa_competence_score

*#> [1] 5*.*5 5*.*0 3*.*0 5*.*0 4*.*0 2*.*5 4*.*5 4*.*5 4*.*5 6*.*0 4*.*5 4*.*0 3*.*5 4*.*0 4*.*0 3*.*5 5*.*0 3*.*5*

*#> [19] 5*.*0 6*.*0 5*.*5 4*.*0 NA 4*.*5 5*.*5 NA 5*.*0 4*.*0 5*.*0 5*.*0 4*.*0 NA 3*.*0 NA 4*.*5 3*.*0*

*#> [37] 6*.*0 NA 1*.*5 3*.*5 4*.*5 6*.*5 2*.*5 5*.*5 3*.*5 6*.*0 4*.*0 6*.*5 5*.*5 5*.*0 6*.*0 NA 3*.*0 5*.*5*

*#> [55] 5*.*0 5*.*0 5*.*5 NA 5*.*0 5*.*0 5*.*0 NA 4*.*5 5*.*0 4*.*0 7*.*0 3*.*5 5*.*5 4*.*5 4*.*5 NA 2*.*5*

*#> [73] 4*.*0 3*.*5 4*.*5 5*.*0 4*.*0 4*.*0 3*.*5 3*.*5 2*.*5 5*.*0 5*.*0 3*.*0 5*.*0 3*.*0 5*.*0 NA 3*.*0 3*.*5*

*#> [91] 3*.*5 5*.*0 5*.*0 NA NA 5*.*0 3*.*5 5*.*0 5*.*0 4*.*0 NA 3*.*5 5*.*0 4*.*5 5*.*5 7*.*5 4*.*0 5*.*0*

*#> [109] NA 4*.*0 6*.*0 NA 5*.*5 6*.*0 5*.*5 3*.*0 4*.*0 3*.*5 4*.*0 6*.*0 3*.*5 4*.*0 NA 5*.*0 6*.*0 7*.*0*

*#> [127] 4*.*0 4*.*5 4*.*5 2*.*5 NA 2*.*5 7*.*5 4*.*5 4*.*0 4*.*0 NA 3*.*0 4*.*0 5*.*5 NA 4*.*0 3*.*5 4*.*5*

*#> [145] 3*.*5 5*.*0 3*.*0 NA 5*.*5 4*.*5 5*.*5 3*.*5 6*.*0 5*.*0 6*.*5 6*.*0 4*.*0 2*.*0 4*.*0 NA 2*.*5 4*.*5*

*#> [163] 4*.*5 5*.*0 6*.*0 5*.*5 2*.*0 7*.*0 NA 3*.*5 3*.*5 NA 5*.*0 NA 5*.*5 6*.*0 5*.*5 5*.*0 4*.*0 4*.*0*

*### For complete output*, *refer to the capl vignette*

#### 4.3.5 Motivation and confidence score

The get_mc_score() function computes a motivation and confidence domain score that ranges from zero to 30 based on the predilection, adequacy, intrinsic motivation and physical activity competence scores. If one of the scores is missing or invalid, a weighted domain score is computed from the other three scores. This score is used to compute the overall physical literacy score.

capl_demo_data$mc_score <- **get_mc_score**(

    predilection_score = capl_demo_data$predilection_score,

    adequacy_score = capl_demo_data$adequacy_score,

    intrinsic_motivation_score = capl_demo_data$intrinsic_motivation_score,

pa_competence_score = capl_demo_data$pa_competence_score

)

capl_demo_data$mc_score

*#> [1] 15*.*333333 20*.*533333 21*.*333333 16*.*400000 NA 14*.*666667 19*.*866667*

*#> [8] 18*.*266667 17*.*200000 20*.*266667 18*.*266667 15*.*866667 18*.*666667 17*.*466667*

*#> [15] 18*.*400000 19*.*466667 18*.*800000 16*.*800000 20*.*533333 21*.*066667 18*.*666667*

*#> [22] 16*.*533333 NA 15*.*600000 22*.*133333 NA 22*.*400000 17*.*600000*

*#> [29] 18*.*133333 22*.*400000 21*.*600000 NA 18*.*800000 NA 23*.*333333*

*#> [36] 14*.*533333 20*.*933333 NA 13*.*466667 13*.*466667 19*.*066667 20*.*000000*

*#> [43] 18*.*000000 22*.*000000 NA 22*.*000000 16*.*000000 23*.*466667 20*.*400000*

*#> [50] NA 20*.*133333 NA 14*.*400000 22*.*000000 15*.*600000 19*.*733333*

*#> [57] 20*.*533333 NA 13*.*866667 15*.*600000 18*.*000000 NA 20*.*000000*

*#> [64] 19*.*600000 18*.*400000 19*.*866667 15*.*200000 17*.*200000 21*.*466667 16*.*533333*

*### For complete output*, *refer to the capl vignette*

#### 4.3.6 Motivation and confidence interpretation

The get_capl_interpretation() function computes an age- and gender-specific CAPL-2 interpretation for a given CAPL-2 protocol or domain score.

capl_demo_data$mc_interpretation <- **get_capl_interpretation**(

    age = capl_demo_data$age,

    gender = capl_demo_data$gender,

    score = capl_demo_data$mc_score,

    protocol = "mc"

)

capl_demo_data$mc_interpretation

*#> [1] "beginning" "progressing" "progressing" "progressing" NA*

*#> [6] "beginning" "progressing" "progressing" "progressing" "progressing"*

*#> [11] NA        "beginning" NA        NA        "progressing"*

*#> [16] "progressing" "progressing" "progressing" "progressing" "progressing"*

*#> [21] "progressing" "progressing" NA        NA        NA*

*#> [26] NA        "progressing" NA        "progressing" "progressing"*

*#> [31] "progressing" NA        "progressing" NA        "achieving"*

*#> [36] NA        NA        NA        "beginning" "beginning"*

*#> [41] "progressing" "progressing" "progressing" "progressing" NA*

*#> [46] "progressing" "beginning" "achieving" NA        NA*

*### For complete output*, *refer to the capl vignette*

#### 4.3.7 Motivation and confidence domain status

The get_capl_domain_status() function computes the status (“complete”, “missing interpretation”, “missing protocol” or “incomplete”) of a CAPL domain.

capl_demo_data$mc_status <- **get_capl_domain_status**(

    x = capl_demo_data,

    domain = "mc"

)

capl_demo_data$mc_status

*#> [1] "complete"        "complete"        "complete"*

*#> [4] "complete"        "missing protocol"      "complete"*

*#> [7] "complete"        "missing protocol"      "complete"*

*#> [10] "missing protocol"     "missing interpretation" "complete"*

*#> [13] "missing interpretation" "missing interpretation" "missing protocol"*

*#> [16] "complete"        "complete"        "complete"*

*#> [19] "complete"        "complete"        "complete"*

*#> [22] "complete"        "missing protocol" "missing interpretation"*

*#> [25] "missing interpretation" "missing interpretation" "complete"*

*#> [28] "missing interpretation" "complete"        "complete"*

*### For complete output*, *refer to the capl vignette*

### 4.4 Knowledge and understanding functions

**Fig 12:**
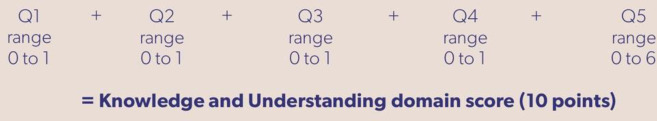
Formula for computing the knowledge and understanding score. Reprinted from www.capl-eclp.ca/capl-manual under a CC BY license, with permission from Healthy Active Living and Obesity Research Group, original copyright 2017.

As illustrated in Fig 12 and in the CAPL-2 manual on page 75 (www.capl-eclp.ca/capl-manual), the formula for computing the knowledge and understanding score is:

#### 4.4.1 Physical activity guideline score (Q1)

The get_binary() function computes a binary score (0 = incorrect answer, 1 = correct answer) for a response to a questionnaire item based on the value(s) set as answer(s) to the item.

capl_demo_data$pa_guideline_score <- **get_binary_score**(capl_demo_data$pa_guideline, **c**(3, "60 minutes or 1 hour"))

capl_demo_data$pa_guideline_score

*#> [1] 0 1 0 0 0 0 1 0 0 0 0 0 0 0 0 0 0 0 0 0 0 0 0 0 0*

*#> [26] 1 1 0 0 0 0 0 0 1 1 0 0 0 0 1 0 0 0 1 0 1 0 0 1 0*

*#> [51] 0 0 0 0 0 0 0 0 1 1 0 0 0 0 0 0 0 1 0 0 0 0 1 0 1*

*#> [76] 0 0 0 1 1 1 0 0 1 0 0 1 0 0 1 0 1 0 0 1 1 0 0 0 1*

*#> [101] 0 0 1 0 1 0 1 0 0 0 0 0 0 0 0 1 0 0 0 1 1 1 1 0 1*

*#> [126] 0 0 0 0 0 1 0 0 0 0 0 1 0 0 1 1 0 0 1 1 1 0 0 0 0*

*#> [151] 0 0 0 0 0 0 1 1 0 0 0 1 0 1 0 0 0 0 0 1 0 1 0 0 0*

*#> [176] 0 1 0 1 0 0 1 1 0 0 0 0 0 1 0 0 0 0 0 0 0 0 0 0 1*

*#> [201] 0 1 0 0 0 0 1 1 0 0 0 0 0 0 1 0 0 0 1 0 0 0 0 0 0*

*#> [226] 0 0 0 0 0 0 1 1 0 0 0 0 0 0 0 1 0 0 1 1 1 0 0 1 0*

*### For complete output*, *refer to the capl vignette*

The get_binary() function is also called to analyze responses for Q2, Q3, and Q4.

#### 4.4.2 Cardiorespiratory fitness definition score (Q2)

capl_demo_data$crf_means_score <- **get_binary_score**(capl_demo_data$crf_means, **c**(2, "How well the heart can pump blood and the lungs can provide oxygen"))

capl_demo_data$crf_means_score

*#> [1] 0 0 0 1 1 0 1 0 0 0 1 1 0 0 0 1 0 0 0 0 0 1 0 0 0*

*#> [26] 0 0 0 0 1 0 0 0 0 0 0 0 0 0 0 0 0 0 0 0 0 0 1 0 0*

*#> [51] 0 0 0 1 0 1 1 1 0 0 0 0 0 0 1 0 0 0 1 1 0 1 1 0 1*

*#> [76] 0 0 0 0 0 1 1 0 0 0 0 0 0 0 0 0 1 0 0 0 0 0 0 0 1*

*#> [101] 1 1 1 1 0 0 0 0 0 0 0 0 0 0 0 0 1 0 1 0 0 0 0 0 0*

*#> [126] 0 0 0 0 0 0 0 1 0 0 0 0 0 0 0 1 1 0 1 1 0 0 0 0 1*

*#> [151] 0 0 0 1 0 1 0 0 0 1 1 1 1 0 0 0 0 0 0 0 0 0 0 0 0*

*#> [176] 0 0 1 1 0 0 0 0 0 0 0 1 1 0 0 0 1 1 1 0 0 0 1 0 0*

*#> [201] 0 0 0 0 0 0 0 0 0 0 0 0 0 0 0 0 0 0 0 0 0 1 0 1 0*

*#> [226] 0 1 0 1 1 0 0 0 0 1 1 0 0 0 0 1 0 0 0 0 0 0 0 0 1*

*### For complete output*, *refer to the capl vignette*

#### 4.4.3 Muscular strength definition score (Q3)

capl_demo_data$ms_means_score <- **get_binary_score**(capl_demo_data$ms_means, **c**(1, "How well the muscles can push, pull or stretch"))

capl_demo_data$ms_means_score

*#> [1] 0 0 1 0 0 1 1 0 0 0 1 1 1 0 1 0 0 0 0 0 0 0 0 0 0*

*#> [26] 0 0 0 0 0 0 0 0 0 0 0 0 0 0 0 0 0 1 0 1 0 0 0 0 0*

*#> [51] 0 1 1 0 1 0 1 1 0 1 0 0 0 0 1 1 1 1 0 1 1 1 0 1 0*

*#> [76] 0 0 0 0 0 0 0 1 0 0 0 0 1 0 0 1 0 1 0 1 0 0 0 0 0*

*#> [101] 1 0 0 0 0 0 0 0 0 0 1 0 0 1 0 1 1 0 0 0 1 1 1 0 0*

*#> [126] 0 0 1 0 1 0 0 0 0 0 0 0 0 0 0 0 0 0 0 0 0 0 1 0 0*

*#> [151] 0 0 0 1 0 1 0 0 1 1 0 0 1 0 0 1 1 0 0 0 0 0 0 1 1*

*#> [176] 1 0 0 0 0 1 1 0 1 0 1 0 0 0 0 0 0 0 0 0 0 0 0 0 0*

*#> [201] 0 0 0 0 0 0 0 0 1 0 0 0 0 0 1 NA 0 0 0 0 0 1 0 1 0*

*#> [226] 0 0 1 0 0 1 0 1 0 0 0 1 0 0 0 1 1 0 0 1 0 1 0 0 0*

*### For complete output*, *refer to the the capl vignette*

#### 4.4.4 Sport skill score (Q4)

capl_demo_data$sports_skill_score <- **get_binary_score**(capl_demo_data$sports_skill, **c**(4, "Watch a video, take a lesson or have a coach teach you how to kick and catch"))

capl_demo_data$sports_skill_score

*#> [1] 0 1 1 0 0 0 0 0 1 0 1 0 0 0 1 0 0 1 1 0 0 0 0 0 0 0 0 1 1 0 0 1 0 0 1 0 0*

*#> [38] 0 0 0 0 0 0 0 0 1 0 1 0 0 0 0 0 0 0 0 1 0 0 0 0 0 0 0 0 0 1 1 1 0 0 0 0 0*

*#> [75] 1 0 0 1 0 1 0 0 0 1 0 0 1 1 0 0 0 0 1 1 0 0 0 1 0 1 0 0 1 1 0 0 1 0 0 1 0*

*#> [112] 0 0 0 1 1 0 0 0 0 1 1 0 0 0 0 0 1 0 0 0 0 1 0 0 0 0 0 1 0 0 0 0 1 0 1 0 0*

*#> [149] 0 0 0 0 0 0 0 0 0 1 0 0 0 0 1 0 0 0 1 0 0 0 1 0 0 0 0 1 0 0 0 0 0 0 0 1 0*

*#> [186] 0 0 0 0 0 1 0 1 0 0 0 0 0 1 0 0 0 0 0 0 0 0 0 0 0 0 1 1 0 1 0 1 0 0 0 0 0*

*#> [223] 0 1 0 0 0 0 0 1 0 0 1 0 0 0 0 0 0 0 0 0 0 0 0 1 0 0 0 0 1 0 0 0 0 0 0 0 1*

*#> [260] 0 0 1 0 1 0 0 0 0 0 0 0 0 1 0 0 0 1 1 1 1 0 0 0 0 0 1 1 0 0 0 0 0 1 0 0 0*

*#> [297] 0 0 0 0 0 0 0 0 0 0 1 0 0 0 1 0 1 0 0 0 0 0 0 0 1 0 0 1 0 0 0 0 0 1 0 0 1*

*#> [334] 0 0 0 1 0 0 0 1 1 0 0 0 1 1 0 0 0 0 0 1 0 0 0 0 0 0 0 0 0 0 0 0 0 0 1 0 0*

*### For complete output*, *refer to the capl vignette*

#### 4.4.5 Fill in the blanks score (Q5)

The get_fill_in_the_blanks_score() function computes a score that ranges from zero to five for responses to the fill in the blanks question. This score is used to compute the knowledge and understanding domain score.

capl_demo_data$fill_in_the_blanks_score <- **get_fill_in_the_blanks_score**(

    pa_is = capl_demo_data$pa_is,

    pa_is_also = capl_demo_data$pa_is_also,

    improve = capl_demo_data$improve,

    increase = capl_demo_data$increase,

    when_cooling_down = capl_demo_data$when_cooling_down,

    heart_rate = capl_demo_data$heart_rate

)

capl_demo_data$fill_in_the_blanks_score

*#> [1] 1 5 2 5 5 3 3 3 3 4 5 4 3 4 4 3 2 2 3 4 3 3 4 1 5 4 6 4 4 4 4 4 1 4 4 2 4*

*#> [38] 4 3 5 4 3 4 3 5 5 4 5 2 3 4 4 2 3 6 3 3 5 5 2 6 2 2 4 2 4 3 3 6 2 3 5 5 3*

*#> [75] 3 2 5 2 3 3 5 4 4 4 5 5 2 3 4 3 4 5 3 1 5 5 5 4 4 4 5 3 4 2 0 3 3 4 4 3 3*

*#> [112] 5 3 4 4 2 2 3 6 4 3 5 5 2 3 3 5 5 3 6 4 3 2 3 3 0 3 4 3 3 3 3 4 3 3 2 5 5*

*#> [149] 4 5 4 3 3 5 0 3 3 4 4 2 2 3 4 1 3 3 2 3 6 3 4 4 4 6 2 4 2 3 2 5 4 3 2 5 5*

*#> [186] 5 3 2 5 3 3 3 3 5 6 2 4 4 3 4 4 2 5 2 4 3 4 4 1 4 3 5 4 3 3 4 3 2 0 5 4 4*

*#> [223] 4 5 3 6 2 5 4 2 3 3 2 4 3 4 3 2 5 3 3 5 3 3 4 5 0 3 4 4 4 4 1 2 4 4 2 3 1*

*#> [260] 3 5 2 3 5 4 1 3 3 3 4 3 3 3 4 3 4 5 0 5 3 3 3 3 5 6 5 3 4 3 3 6 6 2 1 2 2*

*#> [297] 3 5 4 4 2 1 1 1 5 2 2 5 4 3 5 4 6 3 5 2 5 3 2 5 4 5 4 4 1 3 4 6 3 3 3 5 5*

*#> [334] 2 3 4 3 5 5 4 4 0 4 3 4 3 3 3 4 2 3 2 3 6 5 3 5 2 4 4 5 3 2 4 3 2 3 3 5 2*

*### For complete output*, *refer to the capl vignette*

#### 4.4.6 Knowledge and understanding score

The get_ku_score() function computes a knowledge and understanding domain score that ranges from zero to 10 based on the physical activity guideline (Q1), cardiorespiratory fitness means (Q2), muscular strength and endurance means (Q3), sports skill (Q4) and fill in the blanks (Q5) scores. If one of the scores is missing or invalid, a weighted domain score is computed from the other four scores. This score is used to compute the overall physical literacy score.

capl_demo_data$ku_score <- **get_ku_score**(

    pa_guideline_score = capl_demo_data$pa_guideline_score,

    crf_means_score = capl_demo_data$crf_means_score,

    ms_means_score = capl_demo_data$ms_means_score,

    sports_skill_score = capl_demo_data$sports_skill_score,

    fill_in_the_blanks_score = capl_demo_data$fill_in_the_blanks_score

)

capl_demo_data$ku_score

*#> [1] 1*.*000000 7*.*000000 4*.*000000 6*.*000000 6*.*000000 4*.*000000 6*.*000000 3*.*000000*

*#> [9] 4*.*000000 4*.*000000 8*.*000000 6*.*000000 4*.*000000 4*.*000000 6*.*000000 4*.*000000*

*#> [17] 2*.*000000 3*.*000000 4*.*000000 4*.*000000 3*.*000000 4*.*000000 4*.*000000 1*.*000000*

*#> [25] 5*.*000000 5*.*000000 7*.*000000 5*.*000000 5*.*000000 5*.*000000 4*.*000000 5*.*000000*

*#> [33] 1*.*000000 5*.*000000 6*.*000000 2*.*000000 4*.*000000 4*.*000000 3*.*000000 6*.*000000*

*#> [41] 4*.*000000 3*.*000000 5*.*000000 4*.*000000 6*.*000000 7*.*000000 4*.*000000 7*.*000000*

*#> [49] 3*.*000000 3*.*000000 4*.*000000 5*.*000000 3*.*000000 4*.*000000 7*.*000000 4*.*000000*

*#> [57] 6*.*000000 7*.*000000 6*.*000000 4*.*000000 6*.*000000 2*.*000000 2*.*000000 4*.*000000*

*#> [65] 4*.*000000 5*.*000000 5*.*000000 6*.*000000 8*.*000000 4*.*000000 4*.*000000 7*.*000000*

*#> [73] 7*.*000000 4*.*000000 6*.*000000 2*.*000000 5*.*000000 3*.*000000 4*.*000000 5*.*000000*

*### For complete output*, *refer to the capl vignette*

#### 4.4.7 Knowledge and understanding interpretation

The get_capl_interpretation() function computes an age- and gender-specific CAPL-2 interpretation for a given CAPL-2 protocol or domain score.

capl_demo_data$ku_interpretation <- **get_capl_interpretation**(

    age = capl_demo_data$age,

    gender = capl_demo_data$gender,

    score = capl_demo_data$ku_score,

    protocol = "ku"

)

capl_demo_data$ku_interpretation

*#> [1] "beginning" "achieving" "beginning" "progressing" "progressing"*

*#> [6] "beginning" "progressing" "beginning" "beginning" "beginning"*

*#> [11] NA        "progressing" NA        NA        "progressing"*

*#> [16] "beginning" "beginning" "beginning" "beginning" "beginning"*

*#> [21] "beginning" "beginning" "beginning" NA        NA*

*#> [26] NA        "progressing" NA        "beginning" "progressing"*

*#> [31] "beginning" "beginning" "beginning" NA        "progressing"*

*#> [36] NA        NA        "beginning" "beginning" "progressing"*

*#> [41] "beginning" "beginning" "progressing" "beginning" "progressing"*

*#> [46] "achieving" "beginning" "achieving" NA        NA*

*### For complete output*, *refer to the capl vignette*

#### 4.4.8 Knowledge and understanding domain status

The get_capl_domain_status() function computes the status (“complete”, “missing interpretation”, “missing protocol” or “incomplete”) of a CAPL domain.

capl_demo_data$ku_status <- **get_capl_domain_status**(

    x = capl_demo_data,

    domain = "ku"

)

capl_demo_data$ku_status

*#> [1] "complete"        "complete"        "complete"*

*#> [4] "complete"        "complete"        "complete"*

*#> [7] "complete"        "complete"        "complete"*

*#> [10] "complete"        "missing interpretation" "complete"*

*#> [13] "missing interpretation" "missing interpretation" "complete"*

*#> [16] "complete"        "complete"        "complete"*

*#> [19] "complete"        "complete"        "complete"*

*#> [22] "complete"        "complete"        "missing interpretation"*

*#> [25] "missing interpretation" "missing interpretation" "complete"*

*#> [28] "missing interpretation" "complete"        "complete"*

*### For complete output*, *refer to the capl vignette*

## 5.0 Overall physical literacy score

The get_capl_score() function computes an overall physical literacy score that ranges from zero to 100 based on the physical competence, daily behaviour, motivation and confidence, and knowledge and understanding domain scores. If one of the scores is missing or invalid, a weighted score is computed from the other three scores.

capl_demo_data$capl_score <- **get_capl_score**(

    pc_score = capl_demo_data$pc_score,

    db_score = capl_demo_data$db_score,

    mc_score = capl_demo_data$mc_score,

    ku_score = capl_demo_data$ku_score

)

capl_demo_data$capl_score

*#> [1] 54*.*28571 76*.*90000 60*.*00000 68*.*80000 79*.*00000 63*.*00000 52*.*00000 71*.*26667*

*#> [9] 58*.*40000 74*.*66667 83*.*14286 67*.*90000 67*.*50000 65*.*10000 54*.*85714 83*.*60000*

*#> [17] 74*.*60000 77*.*10000 68*.*40000 78*.*80000 75*.*00000 71*.*40000 70*.*83333 64*.*42857*

*#> [25] 68*.*10000 80*.*46667 83*.*30000 68*.*20000 74*.*10000 71*.*30000 65*.*60000 51*.*50000*

*#> [33] 65*.*60000 69*.*23333 79*.*33333 77*.*40000 74*.*20000 80*.*00000 77*.*10000 61*.*10000*

*#> [41] 69*.*30000 81*.*50000 68*.*00000 84*.*50000 81*.*42857 73*.*00000 52*.*00000 86*.*38095*

*#> [49] 64*.*30000 68*.*93333 70*.*10000 83*.*33333 74*.*40000 75*.*50000 79*.*86667 82*.*30000*

*#> [57] 62*.*90000 79*.*20000 86*.*13333 71*.*70000 82*.*50000 67*.*80000 59*.*50000 68*.*14286*

*#> [65] 65*.*80000 79*.*40000 32*.*90000 76*.*30000 71*.*60000 64*.*14286 73*.*04762 67*.*70000*

*#> [73] 71*.*20000 69*.*71429 67*.*50000 75*.*60000 69*.*40000 47*.*40000 60*.*00000 51*.*28571*

*### For complete output*, *refer to the capl vignette*

### 5.1 Overall physical literacy interpretation

The get_capl_interpretation() function computes an age- and gender-specific CAPL-2 interpretation for a given CAPL-2 protocol or domain score.

capl_demo_data$capl_interpretation <- **get_capl_interpretation**(

    age = capl_demo_data$age,

    gender = capl_demo_data$gender,

    score = capl_demo_data$capl_score,

    protocol = "capl"

)

capl_demo_data$capl_interpretation

*#> [1] "progressing" "excelling" "progressing" "achieving" "achieving"*

*#> [6] "progressing" "beginning" "achieving" "progressing" "excelling"*

*#> [11] NA         progressing" NA        NA        "progressing"*

*#> [16] "excelling" "achieving" "excelling" "progressing" "excelling"*

*#> [21] "achieving" "achieving" "achieving" NA        NA*

*#> [26] NA        "excelling" NA        "achieving" "achieving"*

*#> [31] "progressing" "progressing" "progressing" NA        "excelling"*

*#> [36] NA        NA        "excelling" "excelling" "progressing"*

*#> [41] "achieving" "excelling" "achieving" "excelling" "excelling"*

*#> [46] "achieving" "progressing" "excelling" NA        NA*

*### For complete output*, *refer to the capl vignette*

### 5.2 Overall physical literacy domain status

The get_capl_domain_status() function computes the status (“complete”, “missing interpretation”, “missing protocol” or “incomplete”) of a CAPL domain.

capl_demo_data$capl_status <- **get_capl_domain_status**(

    x = capl_demo_data,

    domain = "capl"

)

capl_demo_data$capl_status

*#> [1] "missing protocol"        "complete"        "complete"*

*#> [4] "complete"        "complete"        "complete"*

*#> [7] "missing protocol"        "complete"        "complete"*

*#> [10] "missing protocol"        "missing interpretation" "complete"*

*#> [13] "missing interpretation" "missing interpretation" "missing protocol"*

*#> [16] "complete"        "complete"        "complete"*

*#> [19] "complete"        "complete"        "complete"*

*#> [22] "complete"        "complete"        "missing interpretation"*

*#> [25] "missing interpretation" "missing interpretation" "complete"*

*#> [28] "missing interpretation" "complete"        "complete"*

*### For complete output*, *refer to the capl vignette*

## 6.0 Data visualization

The capl package makes use of the famous ggplo2 R package to create custom functions that render beautiful plots for visualizing CAPL-2 results.

### 6.1 Plots

**Fig 13:**
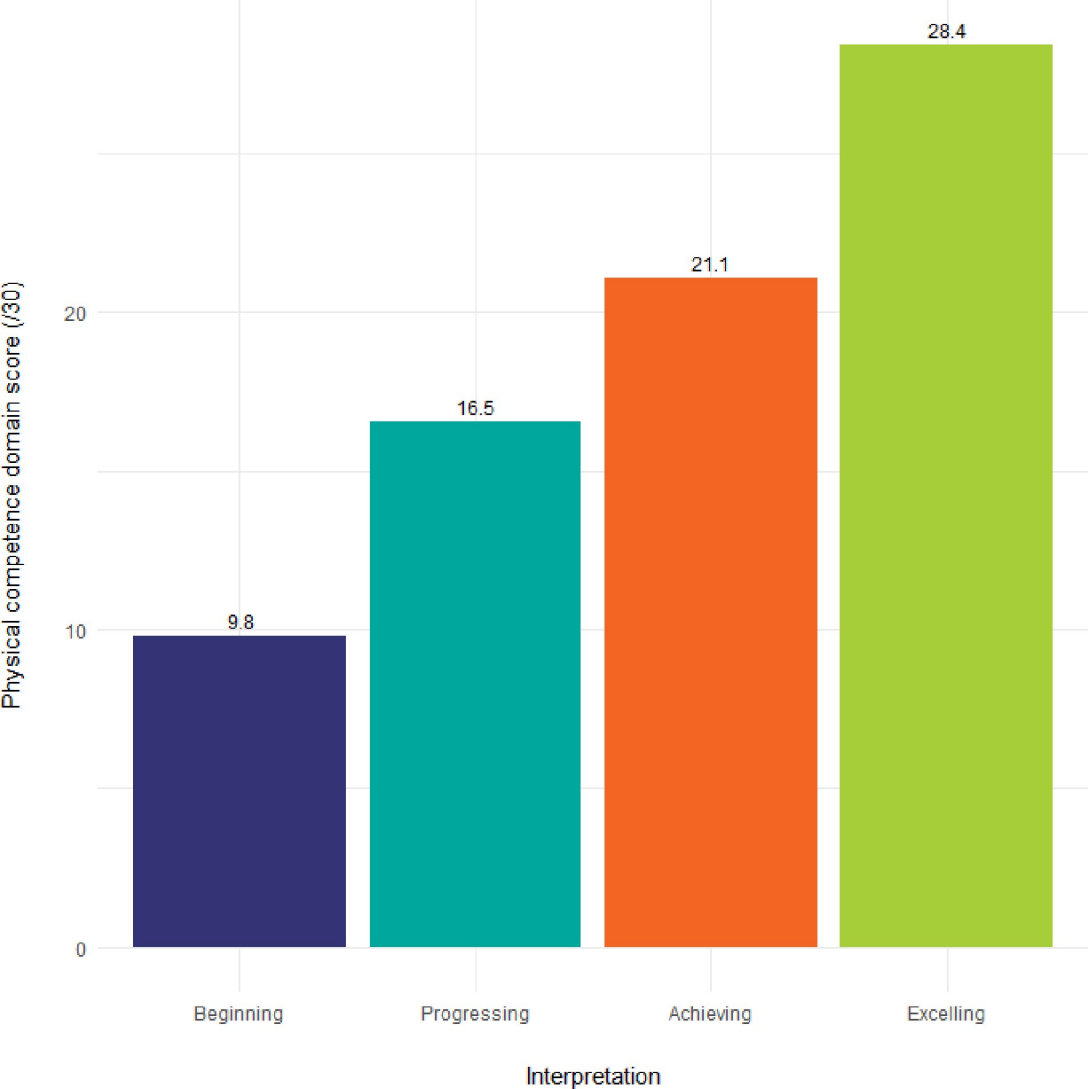
Visual representation of mean scores for each interpretative category using default colours.

CAPL-2 scores can be grouped by their associated interpretative categories and visualized in a bar plot by calling the get_capl_bar_plot() function. The mean score for each interpretative category appears above each bar (see Fig 13).

**get_capl_bar_plot**(

    score = capl_results$pc_score,

    interpretation = capl_results$pc_interpretation,

    x_label = "Interpretation",

    y_label = "Physical competence domain score (/30)"

)

**Fig 14:**
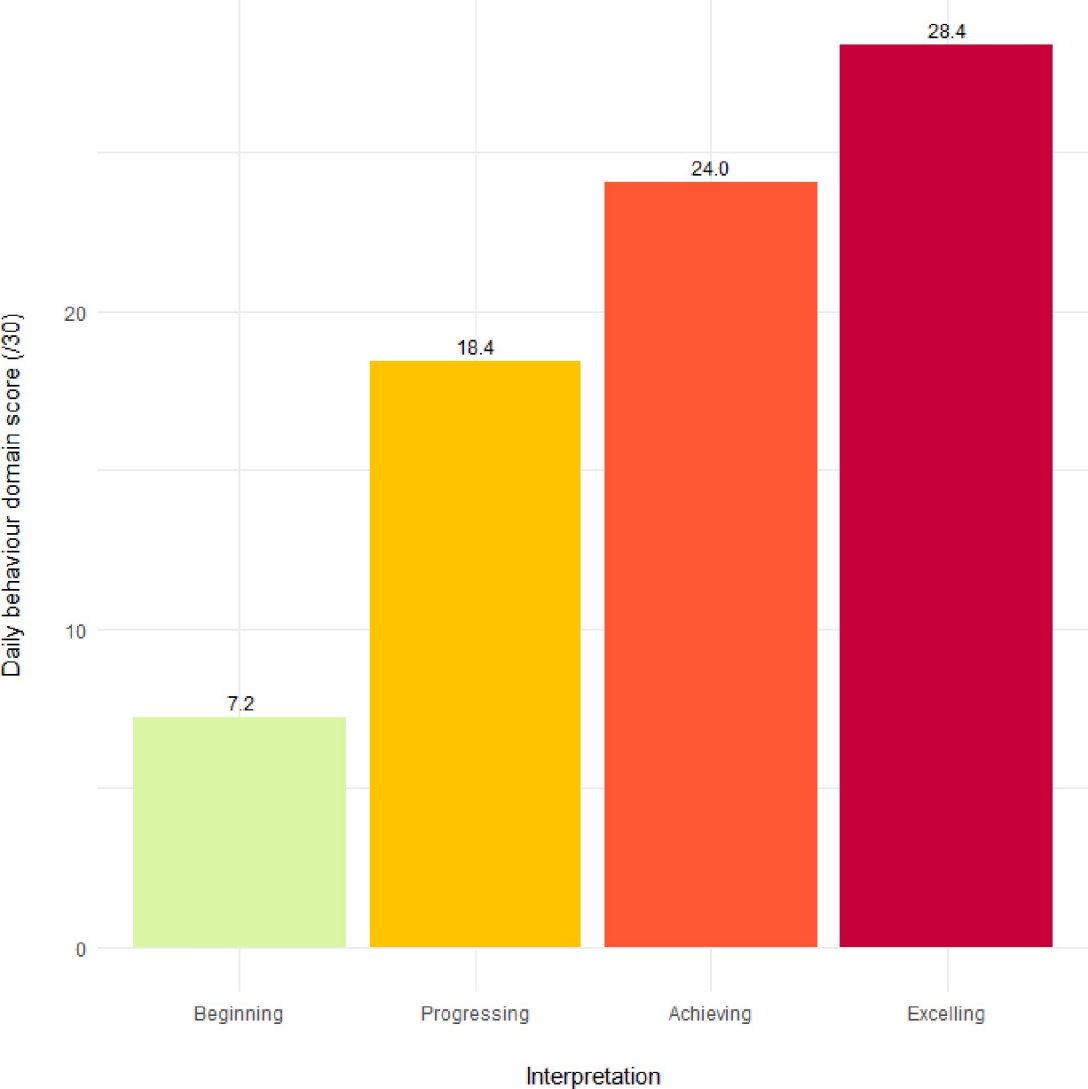
Visual representation of mean score for each interpretative category using custom colours.

The color palette can be customized by setting the colors argument (see Fig 14).

**get_capl_bar_plot**(

    score = capl_results$db_score,

    interpretation = capl_results$db_interpretation,

    x_label = "Interpretation",

    y_label = "Daily behaviour domain score (/30)",

    colors = **c**("#daf7a6", "#ffc300", "#ff5733", "#c70039")

)

## 7.0 Export results

If users want to export their data, the export_capl_data() function allows them to export their data to Excel or SPSS.

**export_capl_data**(

    x = capl_results,

    type = "xlsx",

    file_path = "c:/users/joel/desktop/capl_results.xlsx"

)

**export_capl_data**(

    x = capl_results,

    type = "spss",

    path = "c:/users/joel/desktop/capl_results.sav"

)

## 8.0 Conclusion

In this paper we introduce the capl package developed for use in the R environment. The primary motivation for developing the capl package was to offer interested users – most likely researchers – a fast, efficient, and reliable approach to analyzing CAPL-2 raw data. We begin this paper by discussing several preparatory steps that are required prior to using the capl package. These steps include preparing, formatting, and importing CAPL-2 raw data. We then use demo data to show that computing the CAPL-2 scores and interpretations is as a simple as executing one line of code. This one line of code uses the main (wrapper) function in the capl package (get_capl()) to compute 40 variables. Next, we introduce each helper function that is called within the main function to explain how to compute individual variables and scores for each test element within the four domains as well as how to calculate an overall physical literacy score. Finally, we show how to visualize CAPL-2 results using the ggplot2 R package.

One limitation of the current capl package is that it is specifically built for CAPL-2 raw data, and therefore not fully accessible to users with earlier versions of the CAPL. In the future, we intend to make the capl package available to users across all versions of the CAPL. The future version of the capl package will also include more data visualization features. We also plan to release an R Shiny application that runs locally in a web browser, providing users with a web-based interface for the capl package (e.g., a form for uploading CAPL raw data into R; a form for downloading CAPL raw and computed data out of R into various output formats [CSV, Excel, SAS, SPSS]; a reactive table that updates on the fly as data are uploaded, sorted or filtered, or as new columns are computed or renamed; reactive plots that update on the fly as data and/or variable selections change).

With the development of the capl package, users are no longer required to perform a large number of computations nor are they burdened with the monotonous task of entering data individually for each participant via the CAPL-2 website. Furthermore, we carefully crafted the package to create a “quiet” user experience, whereby “noisy” error messages are suppressed via validation. Instead of throwing noisy errors that halt code execution, the capl package returns missing or invalid values as NAs. The release of the capl package will contribute to the growing and popular topic of physical literacy, and will not only support current users of CAPL-2 but may also attract new users to this area of research.
